# Optimization of Electric Vehicle Drivetrain Fluid with a New System-Level Approach

**DOI:** 10.1080/10402004.2025.2488799

**Published:** 2025-05-19

**Authors:** Joseph F. Shore, Amir Kadiric

**Affiliations:** Imperial College London, London, UK

**Keywords:** Electric vehicle EV, e-fluid, efficiency, transmission, friction, churning

## Abstract

This paper uses a newly developed tribology-based system-level transmission efficiency model to investigate the influence of e-fluid properties on electric vehicle (EV) drivetrain losses. The model considers gear meshing losses using a thermally-coupled mixed friction prediction, bearing losses using existing models, and gear churning using a new experimentally-derived regression equation. The key advantages of the approach are: (i) it is a system-level approach that accounts for the interdependency of different sources of losses by predicting the evolution of temperature distribution in the entire electric drive unit (EDU) including the transmission, e-motor and heat exchanger; (ii) it can discriminate between two oils of the same specification in terms of their impact on overall losses by using measured lubricant rheology; and (iii) it predicts total energy loss over any vehicle duty cycle. The model is validated by comparing its temperature predictions to in-situ measurements made on a real EV in a series of road tests. Application of the model to a typical modern EV shows that it is possible to identify an optimum e-fluid viscosity for minimum transmission losses over any given drive cycle. The exact value of this optimum strongly depends on vehicle duty: it is higher for a city cycle such as the New York City Cycle (NYCC), which has low average speed and frequent start-stops, conditions where gear tooth friction is shown to dominate, and lower for highway driving or the worldwide harmonized light-duty vehicles test cycle (WLTC), where bearing losses dominate. The presented approach provides an efficient tool for optimization of lubricant selection and EDU design.

## Introduction

Properties of the electric drive unit (EDU) lubricant (e-fluid) have an important influence on electric vehicle (EV) power losses and hence driving range. However, owing to the modern EDU being a relatively new mechanical system, there is currently no consensus on the optimum set of fluid properties. Design of an e-fluid is onerous due to competing requirements of different components and the wide range of EDU operating conditions. In particular, recent trends to reduce viscosity, largely driven by attempts to reduce lubricant churning losses and improve cooling at high-speeds, are counterbalanced by requirements to ensure adequate lubricant film thickness to reduce friction and protect gear and bearing surfaces under low-speed, high-torque conditions, which call for higher viscosities. Most modern EDUs implement wet motor designs, where one fluid lubricates the transmission and cools the e-motor, as they provide superior motor cooling compared to dry motor designs. (*1,2*) This imposes additional, and very important, requirements in terms of e-fluid thermal properties and copper compatibility. Given these challenges, it is not surprising that the formulation of e-fluids is subject to extensive research and rapid new developments. To aid these efforts there is a strong need to develop new tools to enable quick and accurate assessment of the impact of different e-fluid properties on overall EDU power losses, as well as to provide some guidelines on the optimum e-fluid viscosity to minimize these losses. This paper attempts to provide such a tool in the form of a tribology-based system-level model to predict the power losses in an EDU over any vehicle duty cycle. To differentiate between two oils of the same specification the model relies on experimental measurements of fluid rheology. The developed tool is then used to systematically investigate the impact of e-fluid viscosity on EDU power losses.

Transmission power losses primarily consist of the load-dependent gear and bearing frictional losses, and the load-independent oil churning and drag losses. Power losses within an EDU will lead to an increase in e-fluid temperature which in turn affects the power losses, with this effect being different for different loss sources. The bearing, gear and churning losses are therefore strongly interdependent through this thermal response of the EDU. In addition, the dynamic nature of torque-speed conditions in an EV transmission imposed by the given vehicle duty cycle induces a continuous temperature evolution of the EDU rather than one steady-state temperature. To be useful in practice, it is therefore crucial for any EV transmission efficiency model to evaluate this temperature evolution using a system-level approach such that it can account for the thermal coupling between the various sources of losses over the course of a complete drive cycle. Such a tool could then provide a holistic assessment of how adjusting a single e-fluid property, such as viscosity, would impact total EDU power losses. An additional challenge in dealing with EV drivetrain tribology are the high transmission input speeds, which often exceed 15,000 rpm; bearing and gear contacts may operate at speeds exceeding those which conventional elastohydrodynamic (EHD) film thickness and traction equations have been tested. (*3–6*) In the context of predicting EDU power losses, the bigger issue is that these speeds are well beyond the validity of commonly used bearing loss models and gear churning regression equations. This must be addressed if an EV transmission efficiency model is to produce valid results.

The current paper presents a tribology-based systems-level EV transmission efficiency model that attempts to address these challenges. The model is then used to investigate the effects of changing oil viscosity on EV transmission losses. The approach considers friction in the gear teeth contacts, churning of the lubricant by the gears, and bearing and seal losses. Its predictions are validated against measurements made on an EV in a series of real-world road tests. Key features of the method are:

- A thermal network approach is implemented to predict the evolution of EDU temperatures and hence account for thermal coupling between the various sources of losses in the transmission, as well as additional heat input from the e-motor and cooling via a heat exchanger.
- Measured lubricant rheology and boundary coefficient of friction (COF) are used as inputs for the prediction of gear friction in an iterative scheme, allowing for nominally similar fluids to be differentiated in terms of their impact on overall transmission efficiency.
- Gear churning losses are predicted with a new regression equation based on new measurements at high gear speeds which are more representative of the high input speeds in an EV transmission.

### Model formulation

This section will describe the estimation of heat transfer essential for predicting thermal coupling between power losses and thereby applying the model to an entire vehicle drive cycle. Next, the determination of each of the individual sources of losses will be described.

### Prediction of EDU temperature evolution

A simple way to estimate transmission temperature evolution is via a lumped mass approach; the gearbox is assumed isothermal, with the predicted temperature representing an average of all the transmission components. This approach was successfully implemented by Christodoulias (*7*) when modeling the power losses of a six-speed manual transmission in an internal combustion engine (ICE) truck, accurately predicting bulk temperature measurements over a real-world drive cycle.

An obvious drawback of this approach is that it neglects the variation of temperature within the gearbox, where different components may operate under different temperatures, with consequent inaccuracies in power loss predictions. Durand De Gevigney et al. (*8*) show that the isothermal assumption inherent in the bulk mass approach was unrealistic for a back-to-back FZG gearbox where temperatures of the gearbox components were predicted to vary significantly from the sump under certain conditions. To address this, several authors (*8–13*) have implemented thermal networks to determine gearbox temperature distribution when predicting power losses. This discretizes the transmission into several isothermal nodes connected by thermal resistances, providing a more granular overview of the temperature variation within the gearbox than the isothermal assumption, without incurring the significant computational cost associated with a full finite element model. Changenet et al. (*9*) modeled the efficiency of a six-speed manual gearbox with this approach. In doing so, localized changes in oil temperature were accounted for when determining gear, bearing, and churning losses using empirical formulae, (*14–16*) and temperature predictions showed good agreement with measurements. The approach was further developed by Durand De Gevigney et al. (*8*) as applied to a back-to-back FZG rig, again showing close agreement to experiments. In typical EV drive units, which often employ wet motor designs, this variation of temperature within the gearbox may be made more significant by the heat transfer between the e-fluid and the e-motor.

**Figure 1.**
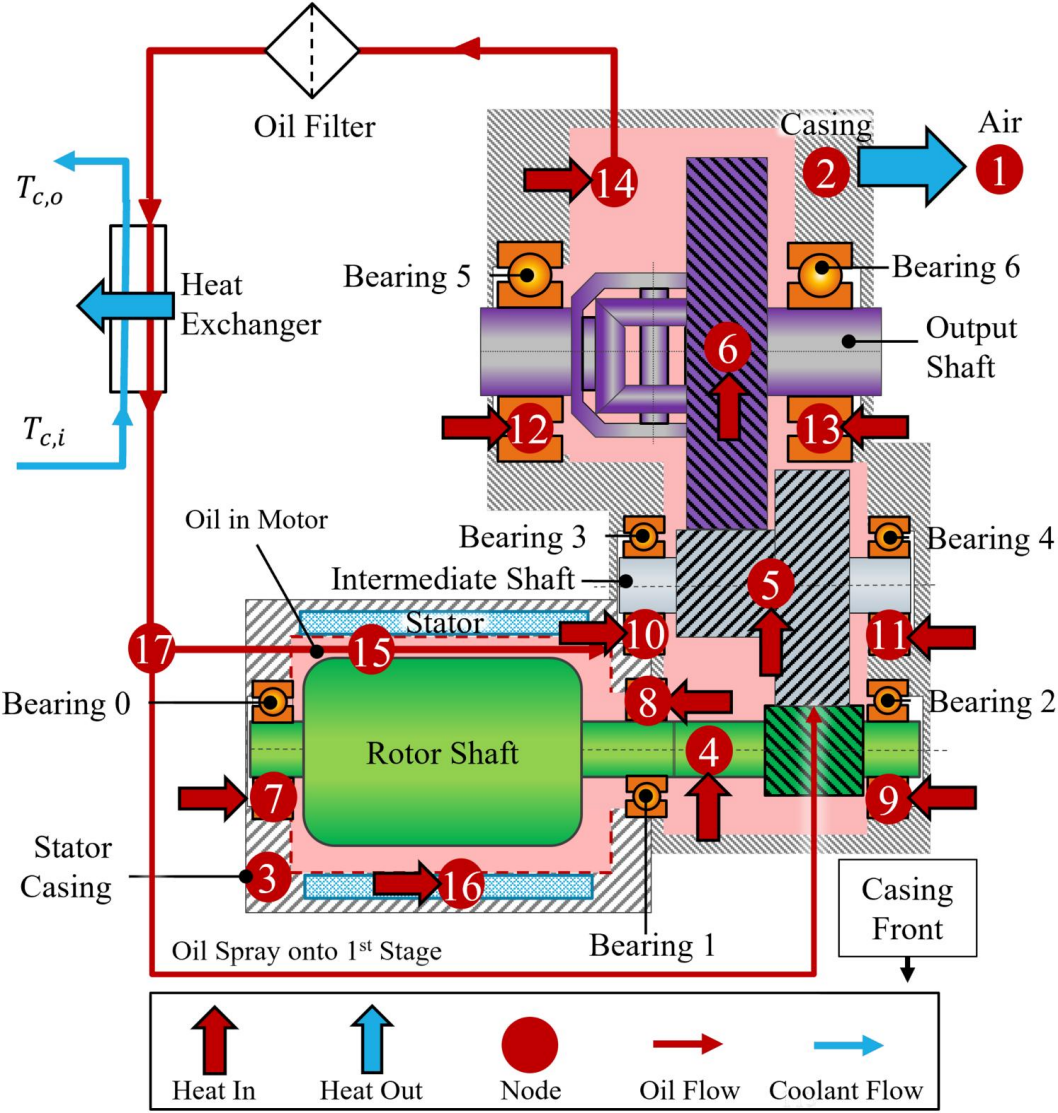
Schematic of the modeled transmission showing oil and coolant flows, heat transfer, and thermal network nodes (numbered red circles).

The present study implements the thermal network approach. The model is applied to a typical EDU, illustrated in the schematic of Fig. 1. This design is representative of the EDUs found in many current EVs. It consists of a two-stage, single speed transmission, with an overall gear ratio of approximately 9:1. Oil from the sump is pumped through a filter, is cooled by a counterflow heat exchanger, and is then split into two streams, one sprayed onto the first gear stage and the other onto the e-motor. While the specifics of the EDU architecture are required for construction of the thermal network, it is important to note that the method is applicable to any EDU design, provided appropriate thermal nodes are used; implementing a different design would require only minor modifications to the modeling procedure.

To model the temperature distribution within this EDU, the present approach implements 17 temperature nodes largely using the general approach of the work by Changenet et al. (*9*) and Durand De Gevigney et al. (*8*) The nodes are indicated by red circles in Fig. 1 and listed in Table A1 in Appendix A, but include all seven bearings, all gears, e-motor stator, oil heat exchanger output and oil sump temperatures. These nodes were connected by thermal resistances estimated using empirical approximations, accounting for heat transfer modes including conduction between the bearings, shafts and casing, centrifugal fling off of oil from the gears, striction through the Hertzian contact between the gear teeth and between rolling elements and raceways in the bearings, as well as the heat transfer coefficients (HTCs) in the motor.

Once these resistances are determined, the network can be represented by a *n*-by-*n* HTC matrix K and the heat into each node Q can be determined from Eq. [1]. The calculation of the HTC matrix and descriptions of each node is provided in Appendix A.

[1] $$Q=\text{KT}+mc_{p}\dot{T}$$

The time derivative vector $mc_{p}\dot{T}$ represents the power loss applied at each node over a 1 s time step of the drive cycle, assuming that the temperature change at each node is instantaneous. The heat capacities ${\left(mc_{p}\right)}_{i}$ of each node were estimated from the material properties and masses of the components as measured from an actual EDU similar to the one in the vehicle. The updated temperature at each node in the gearbox is calculated from the inverse of the HTC matrix. The boundary conditions of the thermal network were taken to be the measured ambient air temperature and the temperature of the oil calculated at the heat exchanger output.

In the present model, any additional heat input into the oil from the e-motor as well as cooling through the heat exchanger is accounted for. The heat exchanger was analyzed using the effectiveness-number of transfer units method, (*17*) with the effective oil and coolant temperatures calculated iteratively. The treatment of the e-motor in this set-up requires further explanation. Heat transfer between the motor and the e-fluid was estimated by approximating the motor geometry as two concentric cylinders, representing the rotor and stator, separated by a long, narrow annulus of oil. HTCs were then estimated using empirical correlations. (*18,19*) It should be noted that the present model does not predict motor losses. Instead, motor power losses were estimated using a publicly available efficiency map for an e-motor of similar characteristics as that used in this EDU. (*20*) The ratio of the motor losses originating in the rotor and stator was assumed to be similar to the results of Wan et al. (*21*) for other permanent magnet synchronous motors. During periods of regenerative braking, the motor efficiency was assumed to be the same as during normal operation. Although the model predicts motor temperatures, as described above, any influence of this temperature on motor losses is ignored because we only have motor efficiency data for a single temperature. This is an important deficiency in the present predictions, but is not a structural deficiency in the actual method—it can easily be overcome if motor efficiency data at other temperatures were available.

### Gear losses

Determination of friction between two meshing gear teeth is complex; contact pressure, lubricant film thickness, film temperature and friction vary continuously along the path of contact. Nevertheless, several empirical gear friction models exist. One of the most well-known is that proposed by Benedict and Kelley, (*14*) appearing in current industrial standards. (*22,23*) Although convenient, it suffers from inaccuracies at low slide-roll ratio (SRRs) near the pitch point. (*24,25*) The presence of the sliding speed in the denominator of its logarithmic term results in the COF prediction tending toward infinity as the contact approaches the pitch point. However, in many practical applications, this discrepancy is acceptable since it has negligible influence on loss predictions, as the COF is multiplied by the sliding speed (which also tends toward zero here) when calculating power loss. (*9*) Some of these shortcomings were addressed by Xu, (*26*) who proposed a formula for gear COF based on around 10,000 results of an elastohydrodynamic lubrication (EHL) model. This successfully predicts the expected reduction in COF to near zero at the pitch point. Although these models can provide reasonable approximations of the operation of the gear pair, the only lubricant parameter considered is the dynamic viscosity; they do not account for the lubricant rheology, so cannot distinguish between two oils of the same viscosity grade but with different formulations. Lubricant rheology strongly influences COF in the gear tooth contacts. This is illustrated by Ziegltrum et al. (*27*) in a comparison between mineral, PAO, and polyglycol oils, showing a significant influence of pressure-viscosity coefficient and limiting shear stress on COF. Clearly viscosity alone is insufficient to accurately predict gear friction.

Here, gear meshing losses are determined by accounting for the coupling between gear tooth bulk temperature, lubricant properties and COF using an iterative procedure. The path of contact is discretized into 200 points to account for the changing contact conditions along the tooth flank. The contact pressure at each of these points is determined using Hertzian formulae, approximating the helical gear teeth geometry with equivalent cones with semi-angle equal to that of the gear’s base helix. The tooth load distribution along the path of contact is approximated with a linear ramp from zero at the start of contact (two pair teeth contact region) to maximum within the single pair teeth contact region. The load profile is assumed symmetrical around the pitch point. Addendum modification coefficients were taken from measurements on the disassembled EDU. EHL traction coefficient is determined using the Johnson and Tevaarwerk (*28*) model based on Eyring rheology, as shown in Eq. [2], which relates strain rate $\dot{\gamma }$ to shear stress τ using a hyperbolic sine function:

[2] $$\dot{\gamma }=\frac{\tau_{0}}{\eta_{p}}\text{sinh}\frac{\tau }{\tau_{0}}+\frac{\dot{\tau }}{G}$$

where $\tau_{0}$ is the Eyring stress of the fluid, and $\eta_{p}$ is the in-contact dynamic viscosity, determined with the Roelands equation (Eq. [3]), at the pressure *p* and temperature *T* of the contact (*29*):

[3] $$\frac{\eta_{p}}{\eta_{\infty }}=\;\text{exp}\;\left[\text{ln}\;\left(\frac{\eta_{r}}{\eta_{\infty }}\right)\cdot {\left(1+\frac{p}{p_{r}}\right)}^{z}\cdot {\left(\frac{T_{r}+135}{T+135}\right)}^{S_{0}}\right]$$

where $\eta_{\infty }$ is a constant, $\eta_{r}$ is measured viscosity at atmospheric pressure and reference temperature $T_{r}\text{,}$ and $p_{r}$ is a reference pressure. (*29,30*) The *z* parameter and the atmospheric slope index $S_{0}$ are lubricant specific and are determined experimentally.

The viscoelastic response term in Eq. [2] $\dot{\tau }/G$ is only significant under very low SRR conditions (*31*) and is thus neglected here. The strain rate $\dot{\gamma }$ is defined as the ratio of sliding speed to the central film thickness $h_{c}\text{,}$ assuming a constant velocity gradient in the central film area. The film thickness is calculated using Chittenden et al.’s regression equations, (*32*) combined with the high-speed correction factor proposed by Hili et al. (*4,5*) This correction accounts for the reduction in film thickness, compared to conventional EHL theory, which has been shown to occur at high-speeds (3–6) due to inlet shear heating. Although EHL film thickness and traction at high speeds is a topic of ongoing research, particularly considering the influences of oil rheology, (*6,33*) power losses in an EDU at speeds where these effects become important (>4 m·s${}^{-1}$ rolling speeds) load-independent sources of losses such as gear churning and bearing drag become more dominant than gear losses in the overall EDU. (*34*)

The model implements the approach of Olver and Spikes (*35*) to calculate the fluid friction for any given condition along the path of contact. The algorithm is fully described in Appendix B and it considers viscoelasticity, shear thinning and if the plastic limit is reached. The end result is the contact mean shear stress $\bar{\tau }$ for the given conditions at any point along the path of contact. $\mu_{f}$ can then be determined using Eq. [4]:

[4] $$\mu_{f}=\frac{\bar{\tau }}{\bar{p}}$$

Although this is sufficient to determine the COF when the gear teeth operate under full-film EHL conditions, gears typically have an appreciable surface roughness and thus frequently operate in the mixed lubrication regime. To account for this, the present approach uses the method proposed by Olver and Spikes, (*35*) where the effective mixed COF is calculated by combining the fluid COF value, determined with the above procedure, and the measured boundary COF $\mu_{b}\text{,}$ using a function which depends upon the specific film thickness (lambda ratio) Λ, as shown in Eq. [5]. This empirical fit estimates the effective mixed COF (the sloping part of the Stribeck curve) from lambda with two asymptotic values: fluid friction $\mu_{f}$ as lambda tends toward infinity, and boundary friction $\mu_{b}$ as lambda tends toward zero. The validity of this approach was later confirmed by the experimental work of Guegan et al. (*36*) The equation was recently adapted by Taylor and Sherrington (*37*) to include an additional constant.

[5] $$\mu =\mu_{f}+\frac{\mu_{b}-\mu_{f}}{{\left(1+\Lambda \right)}^{m}}$$

*m* is a fitting parameter equal to 2. The μ value calculated with Eq. [5] is used except when the point of contact is very close to the pitch point (taken to be when the SRR is below 0.001 here); in this region the effective friction is set to equal fluid friction $\mu_{f}\text{,}$ which is close to zero here anyway (and is exactly zero at pitch point) owing to very low sliding speeds.

### Thermal coupling in the gear mesh

**Figure 2.**
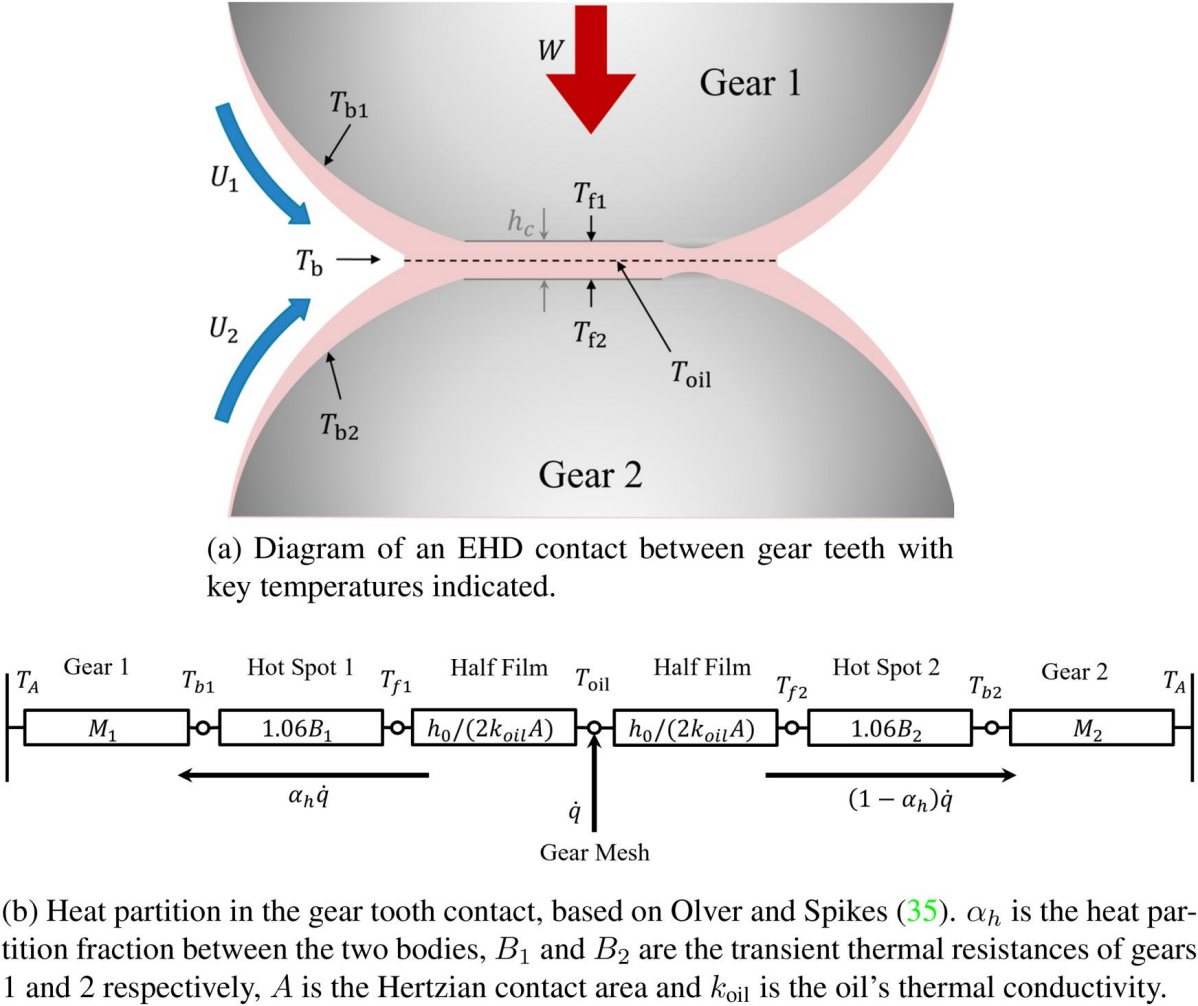
(a) Key temperatures in the gear tooth contact, and (b) thermal network describing the heat partition between the two bodies.

Figure 2a shows a schematic of an EHL contact between a pair of gear teeth. *W* is the applied load, $h_{c}$ is the central oil film thickness, and $U_{1}$ and $U_{2}$ are the surface speeds of gears 1 and 2 respectively. The heat flows within the gear tooth contact (or any other similar EHL contact) are illustrated in the thermal network diagram in Fig. 2b. This accounts for the division of the generated frictional heat between the two gear teeth $\dot{q}\text{,}$ the oil film temperature, $T_{\text{oil}}\text{,}$ tooth surface flash temperatures, $T_{f1}$ and $T_{f2}$ and the gear bulk temperatures, $T_{b1}$ and $T_{b2}\text{.}$ The temperature of the surroundings in which the gear operates (the oil-air mixture) is $T_{A}\text{.}$ Two key temperatures are required to determine friction in the gear tooth contact: the oil temperature at the inlet $T_{b}\text{,}$ required for determining the lubricant viscosity used to calculate the film thickness $h_{c}\text{,}$ and the in-contact temperature $T_{\text{oil}}\text{,}$ which is used to determine the lubricant properties for the friction predictions. Frictional heat input in the gear tooth contact increases the gear bulk temperatures, which increase the oil inlet temperature ($T_{b}$) and in turn reduces the oil inlet viscosity and hence the oil film thickness; this then affects the friction in the contact and therefore the frictional heat generated. These in-contact oil temperatures, oil inlet temperature, film thickness and COF are thermally coupled, and therefore their determination requires an iterative approach.

**Figure 3.**
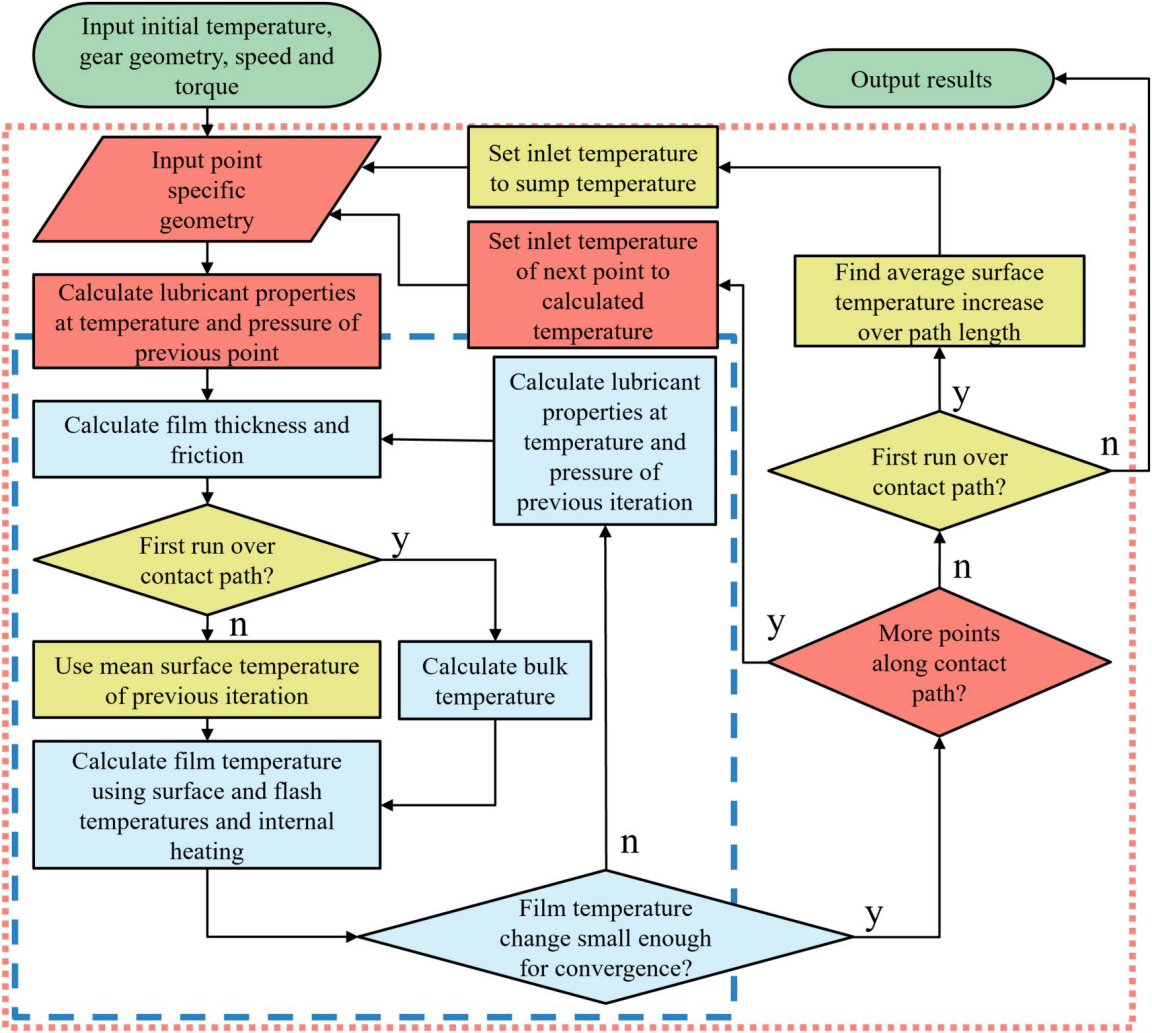
Flowchart showing the iterative procedure used to determine the gear tooth contact friction. Inner loop (blue, dashed line) is used to predict oil film thickness, oil film temperature, gear tooth temperature and gear tooth contact friction at a single point, and the outer loop (red, dotted line) to step along the entire path of contact during the mesh cycle. Yellow indicates second run over path of contact.

Figure 3 shows a flowchart of the iterative procedure used here to determine the relevant temperatures and hence the gear friction losses at each point along the contact path, accounting for thermal coupling between friction, temperature, and lubricant properties. The inner loop of the procedure, indicated with blue flowchart symbols and the broken line in Fig. 3, iteratively determines the temperature, lubricant properties, film thickness and friction at a single point along the path of contact.

The mean fluid film temperature is equal to the bulk temperature $T_{b}\text{,}$ plus the flash temperature rise of either surface $\Delta T_{f}$ plus any additional temperature rise across the fluid film itself $\Delta T_{\text{oil}}$ (the latter is only significant in thick oil film condition), as expressed by Eq. [6]:

[6] $$T_{\text{oil}}=T_{\text{bi}}+\Delta T_{\text{fi}}+\Delta T_{\text{oil}}$$

where *i* is either 1 or 2, corresponding to gear 1 or gear 2. The procedure is considered converged when the discrepancy between the calculated fluid film temperature and that used to determine the lubricant properties is less than 0.5${}^{{}^{\circ}}$C. This procedure is nested within the outer loop, indicated in red in Fig. 3, which increments along the path of contact; the converged properties from the inner loop act as the starting values for the subsequent point. Finally, once the procedure has reached the end of the path of contact, it is repeated. In this second run, as indicated in yellow, the bulk temperature of the gear tooth is taken as the mean of the values calculated at each point in the first. Since the path of contact is traversed quickly, the bulk thermal mass of the gear tooth will prevent rapid changes in its temperature; thus, this average value represents a more realistic estimate of gear bulk temperature.

The approach requires the determination of steady-state gear thermal resistances ($M_{1}$ and $M_{2}$) to determine the gear bulk temperatures, requiring knowledge of the HTC between the gear bulk and the surroundings. This HTC is difficult to establish accurately for a rotating gear due to the complexities of gear geometry and the surrounding two-phase mixture of oil and air. To calculate this accurately a multiphysics computational fluid dynamics (CFD) approach is needed and some aspects of this have been explored by the present authors. (*38*) However, this is a computationally expensive approach and is therefore not an option in the present model which aims to consider the evolution of losses over the entire drive cycle and hence requires a computationally efficient method. Instead, in the present work this HTC is estimated following the approximation described by Olver (*39*) where the heat transfer from the gear sides is calculated by considering the gear as a short cylinder and applying corresponding standard heat transfer solutions, and the heat transfer from the gear track is calculated using similar solutions for a long cylinder.

### Churning losses

**Figure 4.**
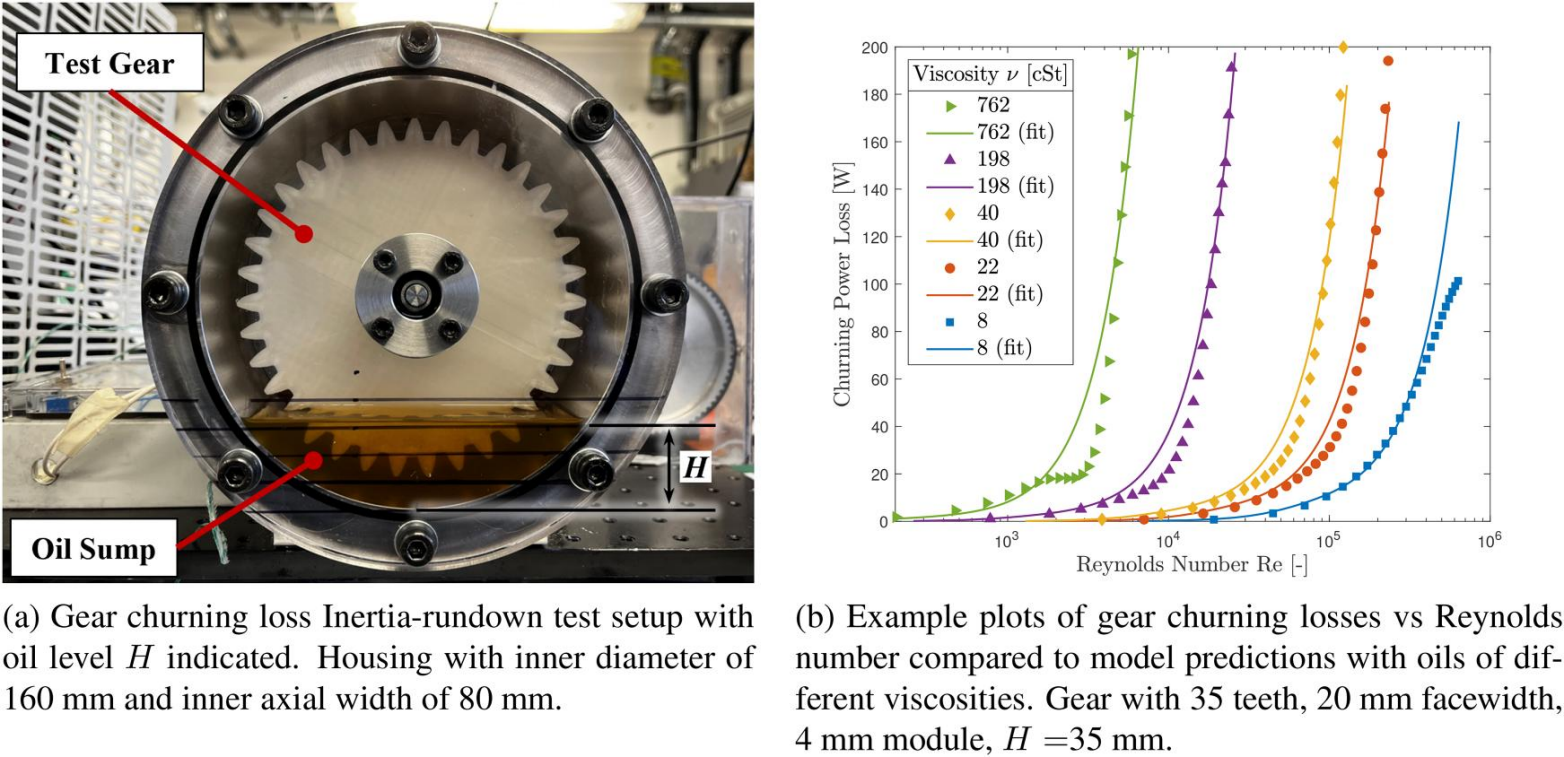
(a) Gear churning inertia rundown test rig, and (b) example results.

Gear churning losses arise from viscous dissipation due to the rotation of a gear through a lubricant sump. This source of loss depends on several factors, including rotational speed, lubricant properties, gear and casing geometry, and gear immersion depth. There are a number of empirically derived churning loss formulae (*16,40–44*) but it is recognized that these models do not always provide accurate predictions and indeed, may predict very different churning losses under certain conditions. (*45*) Unfortunately, these existing regression equations for gear churning do not cover the high gear speeds commonly encountered in EV transmissions. To overcome this issue, in this work we predict churning losses with a newly derived regression formula based on new churning loss measurements that cover speeds representative of EV transmissions. The formula is based on the approach of Shore et al. (*46*) but with extension to higher gear speeds. The formula is based on extensive measurements of gear churning torque using an inertia rundown method with several oil viscosities, gear geometries, casing geometries, immersion depths and gear speeds of up to 90 m ·s${}^{-1}$ pitch line velocities (12,000 rpm for gear diameters used in the tests). The set-up, details of test procedure and derivation of regression fits are fully described in Shore et al. (*46*) and in Shore (*47*) specifically for high speeds, but a brief description is provided here for convenience. A photograph of the inertial rundown experimental setup is shown in Fig. 4a and example results are shown in Fig. 4b.

The churning loss regression equation accounts for the influences of oil kinematic viscosity ν, density ρ, the gear’s tip $R_{a}$ and reference $R_{p}$ radii, facewidth *b*, rotational speed ω, and size of the cylindrical casing (*via* axial δx and radial δr clearances). The final regression equation used here is shown in Eq. [7]:

[7] $$C_{\text{ch}}=0.38\rho \nu^{0.05}R_{a}^{8.51}R_{p}^{-5.90}b^{0.20}\delta r^{0.08}\delta x^{0.34}h_{\text{eff}}^{1.10}\omega^{0.81}g^{0.57}$$

where *g* is gravitational acceleration (9.8 m·s${}^{-2}$); and $h_{\text{eff}}$ represents the “effective” immersion depth parameter, which accounts for the reduction in immersion depth due to the distribution of oil around the casing by the rotating gear, which strongly depends on speed and viscosity. This is defined by Eq. [8]:

[8] $$h_{\text{eff}}=h_{0}\left[\phi +(1-\phi )\text{tanh}\left\{0.2+\frac{{\text{Re}}^{0.4}}{{\text{Fr}}^{1.0}}\right\}\right]$$

where $h_{0}$ is the nominal (initial) immersion depth and ϕ is the fill ratio of the gear casing, equal to 1 when the casing is completely filled. Re and Fr are the Reynolds ($=\omega R_{p}^{2}/\nu$) and Froude ($=\omega^{2}R_{p}/g$) numbers, respectively.

The use of this effective immersion follows the method presented by Shore et al. (*46*) and its inclusion is important in fully describing the relationship between gear churning loss and oil viscosity. It may intuitively be expected that churning losses increase with increasing oil viscosity. However, recent work by Shore et al. (*46*) shows that this is not always true and that in fact higher viscosity can lead to lower churning loss under some conditions. This is because as a gear rotates, it distributes the oil from the sump around the casing thus reducing the effective immersion depth; the amount of reduction is determined by the balance of flow of oil back into the sump and it being thrown up by the rotating gear, and as such is often more significant for a higher viscosity oil. Since immersion depth has a stronger influence on churning losses than viscosity itself (as indicated by the larger exponent on the immersion depth term in many churning loss models (*16,40–44*)), this can result in a higher viscosity fluid producing lower churning losses than a lower viscosity one under certain conditions.

The present model was developed from experiments on a single gear. However, it is known that in a gear pair, the gear rotational sense can strongly influence churning losses due to effective increase in immersion depth in the vicinity of the pinion due to the combined motion of the two gears, referred to as “swell effect.” (*16*) To account for this additional churning loss in a gear pair, the corresponding additional term proposed by Changenet and Velex (*16*) has been applied in the present churning loss model wherever needed when considering a full EV transmission.

### Bearing losses

Losses in rolling element bearings comprise both load-dependent and load-independent sources. The former are due to frictional losses in the rolling element-ring contacts, and the latter are due to lubricant drag and seal losses. There exist several bearing loss models, one of the most widely used is the SKF “four sources” model, (*48,49*) in which the total bearing loss is the sum of four components: sliding frictional losses, hydrodynamic oil losses due to rolling, oil drag and seal losses. However, the model has limitations for application to EVs; SKF stipulate that it should not be used when the $n\cdot d_{m}$ speed parameter, (where *n* is the bearing rotational speed and $d_{m}$ is the mean bearing diameter) exceeds $0.5\times 10^{6}$ mm·rpm. (*50*) At higher speeds the model significantly overpredicts the losses, primarily due to the overestimate in drag moment, which increases with the square of rotational speed. (*51*) In current and future EVs, bearings on the input shaft may regularly exceed this limit at normal road speeds (*34,47*) such that a direct application of this model to predict bearing losses in an EV transmission can lead to inaccurate results over many vehicle duty cycles.

More recently, Morales-Espejel and Wemekamp (*50*) adapted the original treatment of the bearing drag losses to better deal with speeds beyond the 0.5$\times 10^{6}$ mm·rpm $n\cdot d_{m}$ limit. The approach attempts to account for the effective properties of the oil/air mixture in the bearing at high speeds so that, rather than monotonically increasing with rotational speed, the bearing drag moment may reduce due to a decrease in effective oil viscosity as it is mixed with air.

The present EV transmission losses model employs the SKF bearing friction model with this updated treatment of drag losses so that it can be used at all vehicle speeds. However, it should be noted that the bearing loss model used here still only provides estimates, particularly since the publicly available model, as implemented here, includes several simplified and estimated factors for ease of use at the expense of accuracy.

To implement the bearing loss model in the present work, the magnitudes of each source of bearing loss were calculated from the operating conditions and bearing geometry using tables provided in the SKF catalog (*49*) and bearing radial and axial loads determined by solving free-body diagrams of each shaft, accounting for component locations and the meshing forces of the helical gears. The difference in speeds of the output shaft bearings during cornering due to the differential has been neglected. However, as the losses from the output shaft bearings are expected to be small relative to the overall transmission losses due to their low speed, (*34*) any error incurred is expected to be insignificant. Determination of the oil level in the bearings of the input shaft is complex since they are located above the nominal sump level; they rely on the distribution of oil during operation for lubrication. In the results presented in this paper, the immersion depth for the bearings on this shaft has been set to zero, that is, drag losses are neglected. The implications of this assumption are examined in the discussion section.

### Experimental lubricant characterization

To make it possible to accurately assess the impact of lubricant properties on EDU power losses and hence differentiate between two oils of the same specification, the EDU power loss model uses measured lubricant traction properties. These are measured following the procedure first described by Lafountain et al. (*52*) In this procedure, a series of traction curves are obtained to cover a large range of contact pressures, temperatures and SRRs. In parallel, the oil film thickness is also measured to obtain the shear rates that exist in the traction tests. This data is then used to extract the Eyring shear stress, $\tau_{0}\text{,}$ and Roelands parameter, *z*.

In this study the extreme traction machine (ETM) ball on disk tribometer (*53*) was used for traction measurements because it can reach higher pressures than the more common mini traction machine (MTM) rig (which is limited to 1.25 GPa with standard AISI 52100 specimens (*54*)), so that the conditions at which the oil is characterized are representative of gear teeth contacts. Measurements were performed at contact pressures ranging from 1.25 to 2.68 GPa, temperatures from 25 to 145 °C, SRR from 1% to 85%, and constant entrainment speed of 2.75 m · s${}^{-1}\text{.}$ The film thickness was measured under the same entrainment speed and temperature using the EHD optical interferometry rig. (*55*) The shear stress and strain rates are then determined from the measured film thickness and COF respectively. The relationship between shear stress and strain rate expressed with Eq. [2] can be approximated with Eq. [9] while $\tau >1.5\tau_{0}\text{:}$

[9] $$\tau =\tau_{0}\;\text{ln}\;\dot{\gamma }+\tau_{0}\;\text{ln}\;\left(\frac{2\eta_{p}}{\tau_{0}}\right)$$

Thus, a plot of shear stress τ against strain rate $\dot{\gamma }$ on semi-log axes is a straight line (prior to the drop in traction due to thermal effects at higher shear rates) with its gradient being the Eyring stress, $\tau_{0}\text{.}$

Once $\tau_{0}$ is determined, the *z* parameter can be obtained by curve-fitting τ to $\dot{\gamma }$ using Eq. [10], derived from Eqs. [2] and [3].

[10] $$\tau =\tau_{0}{\text{sinh}}^{-1}\left\{\frac{\dot{\gamma }}{\tau_{0}}\text{exp}\;\left[\text{ln}\;\left(\frac{\eta_{r}}{\eta_{\infty }}\right){\left(1+\frac{p}{p_{r}}\right)}^{z}{\left(\frac{T_{r}+135}{T+135}\right)}^{S_{0}}\right]\right\}$$

Boundary COF $\mu_{b}$ for each oil of interest also needs to be measured as input to the model. This is needed to calculate the COF in the mixed regime as specified in Eq. [5]. Boundary COF is determined from a Stribeck curve measured on a standard MTM ball-on-disk rig, but with rougher (Rq≈650 nm) than standard disks so that boundary regime can be reached. Several additional lubricant parameters were measured to fully characterize the test lubricants, both to determine the COF in the gear contacts as well as heat transfer within the gearbox. Descriptions of the experimental methods used are excluded here for brevity but are described in detail in Shore. (*47*)

### Model validation with real-world EV drive cycles

To validate the model’s predictions, they were compared to measurements made on a real popular modern EV during a series of real-world tests, both on the road and on a dynamometer. The estimated gear parameters and bearing designations are summarized in Tables 1 and 2, respectively. Since it is not possible to measure actual transmission power losses in a moving vehicle, the comparison was made between predicted and measured temperature evolution at multiple locations within the EDU during the drive cycles. The temperature rise is a direct consequence of the power losses so that this temperature comparison is an indirect way to compare the predicted and actual EDU power losses. Many tests were performed with substantially different vehicle duties to ensure that the thermal network is not overfitted to a particular set of conditions. This section describes two such tests. Additional test data can be found in Shore. (*47*)

**Table 1.** Estimated gear parameters.

		Stage 1		Stage 2	
Parameter	Units	Pinion	Wheel	Pinion	Wheel
Total gear ratio	[-]	9.036			
Gear ratio $z_{2}/z_{1}$	[-]	2.613		3.458	
Tooth number *z*	[-]	31	81	24	83
Module $m_{n}$	[mm]	1.7		2.6	
Facewidth *b*	[mm]	30		46	
Profile shift *x*	[-]	0.12	0.12	0.16	0.04
Helix angle β	[${}^{{}^{\circ}}$]	20		22.5	

**Table 2.** Bearing designations.

Bearing	0	1	2	3	4	5	6
Designation	6007	6208	6308	6308	6308	6309	6211

### Vehicle experimental setup

Temperatures were measured in several locations, situated so that they may coincide with different nodes in the thermal network employed by the model. Temperatures were measured using a combination of contact thermocouples (placed on the exterior of the drive unit), probe type thermocouples in the oil sump and heat exchanger, and using the vehicles internal instruments as read from the controller area network (CAN) bus for the coolant and stator temperature. The locations at which temperatures were measured and their corresponding nodes in the thermal network are summarized in Table 3.

**Table 3.** Locations and methods of temperature measurement on the drive unit.

Description	Node	Source
Ambient air	1	Thermocouple outside casing
Casing rear	2	Touch thermocouple
Casing front	2	Touch thermocouple
Casing bottom	2	Touch thermocouple
Casing sump level	2	Touch thermocouple
Stator casing bottom	3	Touch thermocouple
Bearing 5	12	Touch thermocouple
Bearing 6	13	Touch thermocouple
Oil sump	14	Thermocouple in oil sump
Stator	16	CAN bus
Heat exchanger oil outlet	17	Thermocouple in heat exchanger
Heat exchanger coolant inlet	–	CAN bus

Four thermocouples were used to measure the temperature of the transmission casing (node 2). This was done to assess whether it was reasonable to represent the entire casing as a single, isothermal node. The touch thermocouples were placed centrally on the front and rear, at the lowest point of the bottom, and on the side of the casing at approximately the nominal sump level. The sump temperature was measured by a thermocouple inserted into the sump through the oil filter. The temperatures of the output shaft bearings were measured using touch thermocouples situated on the exterior of the casing adjacent to the outer race of the bearings. The transmission fluid used in these road tests was characterized using the procedures described above and used as input to the model in these comparisons. A summary of the nominal fluid properties is shown in Table 4.

**Table 4.** Nominal test oil properties.

Parameter	Units	Value
Kinematic viscosity at 40 °C	[cSt]	27.3
Kinematic viscosity at 100 °C	[cSt]	5.9
VI	[-]	174
Density at 20 °C	[kg·m${}^{-3}$]	839
Thermal conductivity at 20 °C	[W·m${}^{-1}$K${}^{-1}$]	0.16
Boundary COF	[-]	0.13

Abbreviation: VI, viscosity index; COF, coefficient of friction.

### Real-world road cycle details

**Figure 5.**
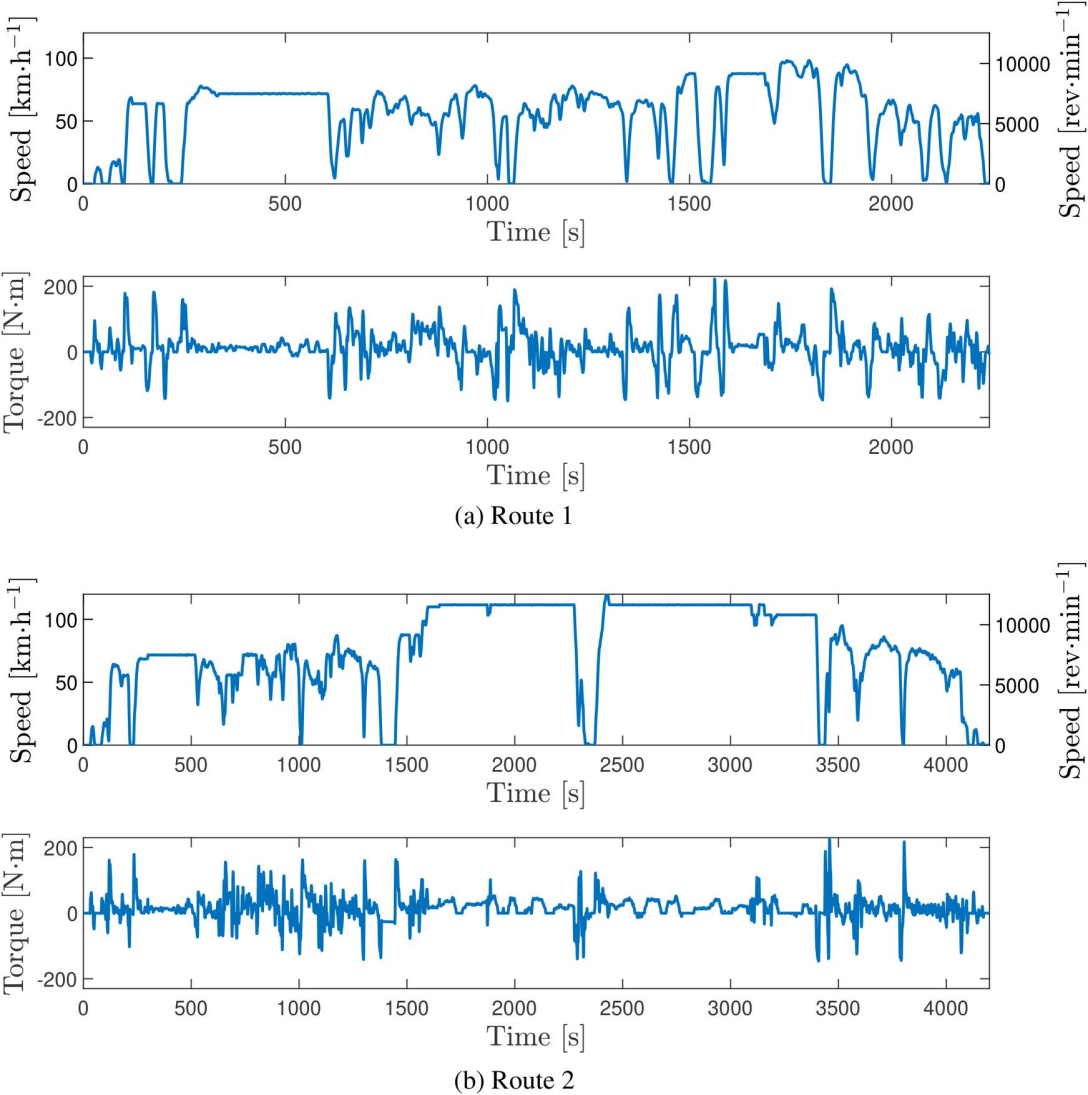
Vehicle road speed and corresponding transmission input speed and torque against time for the two experimental drive cycles. (a) Route 1, (b) Route 2.

Two routes were driven when road testing the vehicle. Route 1 (Fig. 5a) consisted of rural driving along winding roads and hills and a short section of moderate speed cruising. The maximum speed on this route was approximately 98 km·h${}^{-1}$ (≈ 60 mph). Route 2 (Fig. 5b) incorporated an extended period of high-speed cruising, reaching a maximum speed of approximately 122 km·h${}^{-1}$ (≈ 75 mph). The motor torque (i.e., transmission input torque) and vehicle speed, as well as the flowrates of oil and coolant through the heat exchanger were taken from the vehicle’s CAN bus. The transmission input torque, road speed, and corresponding transmission input speed, for the two routes are shown in Fig. 5. The characteristics of the two routes are also summarized in Table 5.

**Table 5.** Summary of experimental road test routes.

Parameter	Units	Route 1	Route 2
Total Time	[s]	2242	4200
Total Distance	[km]	34	89
Top Speed	[km·h${}^{-1}$]	98	122
Mean Speed	[km·h${}^{-1}$]	55	76

### Comparison of model predictions to experimental temperature measurements

**Figure 6.**
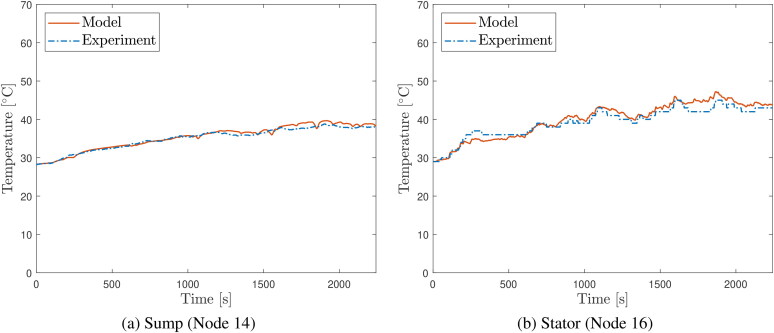
Comparison of temperature evolution predictions to thermocouple measurements over the Route 1 road test for (a) the electric drive unit (EDU) oil sump temperature (node 14) and (b) e-motor stator (node 16).

**Figure 7.**
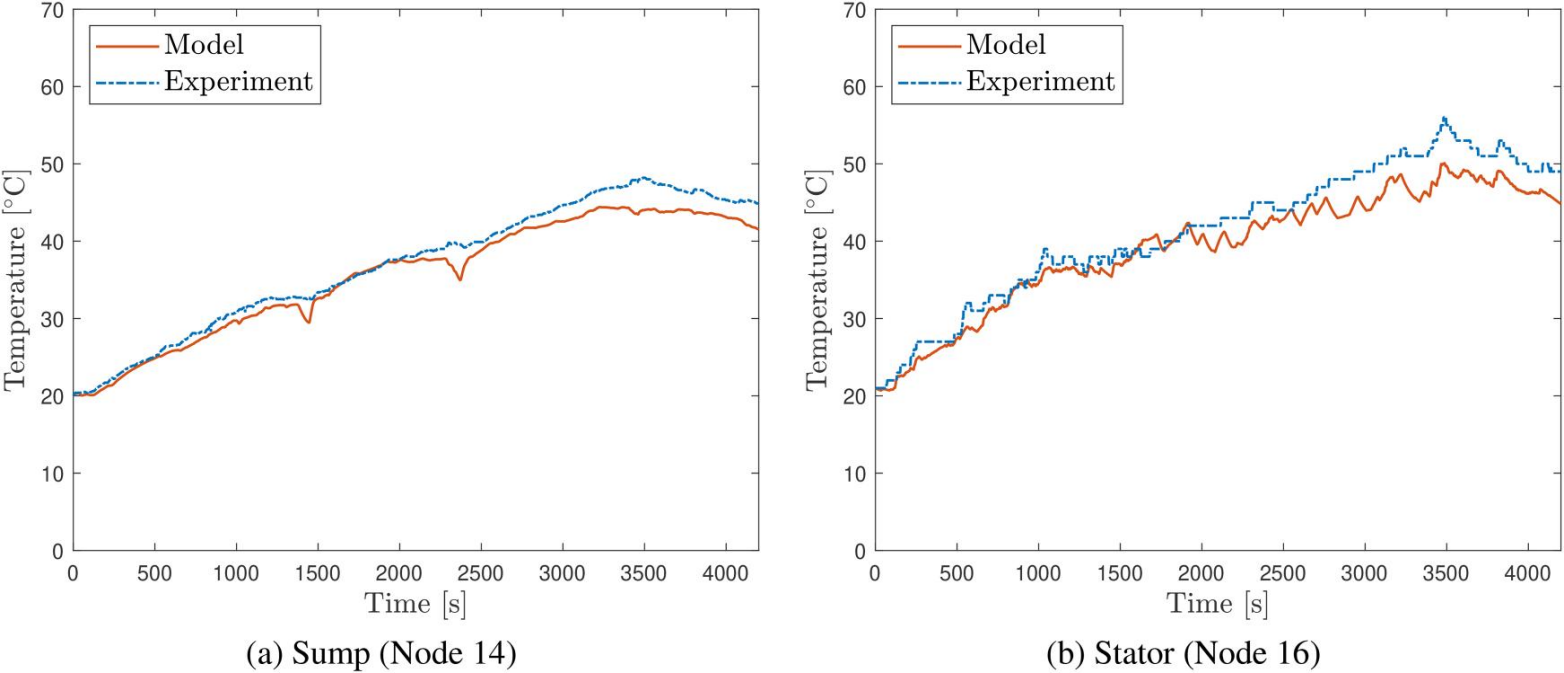
Comparison of temperature evolution predictions to thermocouple measurements over the Route 2 road test for (a) the electric drive unit (EDU) oil sump temperature (node 14) and (b) e-motor stator (node 16).

Figures 6 and 7 show comparisons of predicted evolutions of oil sump and stator temperatures to measurements on the vehicle during the course of the two experimental drive cycles. Similar comparative plots for the other temperature nodes are given in Appendix C. Generally, the temperature evolution at all nodes is predicted well, closely matching measured temperature values as well as the undulations in these values over the course of the road tests. The ability of the model to predict temperature over different vehicle duties is a good indication that the thermal network approach is robust and is not overfitted to a particular duty cycle.

Nevertheless, there are some discrepancies of note between the predictions and the measurements; Fig. 7b shows a slight divergence between the stator temperature predictions and the measurements as the tests progress. Divergence may be expected as any errors over the course of the cycle will accumulate, leading to the predictions drifting from the experimental data over time. The touch thermocouples on the exterior of the casing consistently measure temperatures slightly below the predicted values, perhaps due to additional modes of heat transfer from the EDU to the rest of the vehicle which are unaccounted for by the model. Nevertheless, general trends are captured with reasonable accuracy.

## Results

### Evolution of gear, bearing, and churning losses over a complete drive cycle

**Figure 8.**
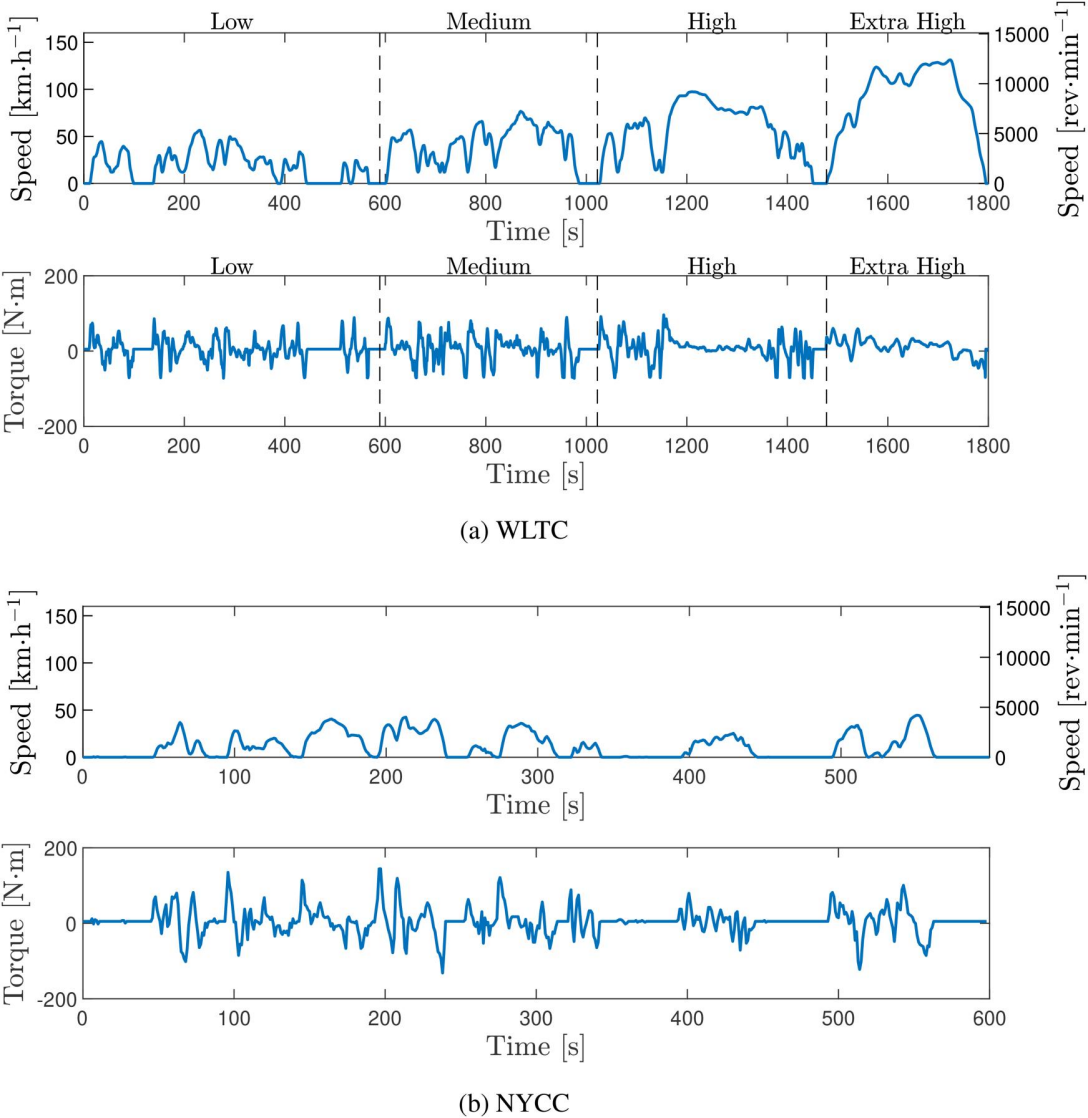
Vehicle road speed and corresponding transmission input speed and torque against time for (a) the class 3 light-duty vehicles test cycle (WLTC) and (b) New York City Cycle (NYCC) vehicle drive cycles.

A principal benefit of the model is its ability to assess the contribution of different loss sources over an entire drive cycle. The model is now used to analyze losses over the two real world experimental drive cycles previously described, as well as for the class 3 worldwide harmonized light-duty vehicles test cycle (WLTC), (*56,57*) widely used for published EV range estimations as outlined by the worldwide harmonized light vehicles test procedure (WLTP), (*57*) and for the New York City Cycle (NYCC), (*58*) which aims to emulate the low-speed, start/stop driving that is typical in large cities. The road speeds and corresponding motor speeds and torques for this vehicle over the WLTC and NYCC are shown in Fig. 8.

Unlike when analyzing the real-world cycles, where torque and speed were taken directly from the vehicle CAN bus, for the WLTC and NYCC torque and speed were instead estimated from the road speed versus time data by considering the vehicle mass, gear ratio, tire radius, drag coefficient, and coefficient of rolling resistance. During periods of deceleration, a braking torque through the EDU was included in the power loss calculations by assuming maximal use of regenerative braking for these motor characteristics. The coolant and oil flowrates and coolant temperature in the heat exchanger were assumed constant and set to representative values recorded by the CAN bus during the real-world road tests previously described. All initial gearbox temperatures were set to the prescribed 23 °C ambient temperature of the WLTP. (*57*)

**Figure 9.**
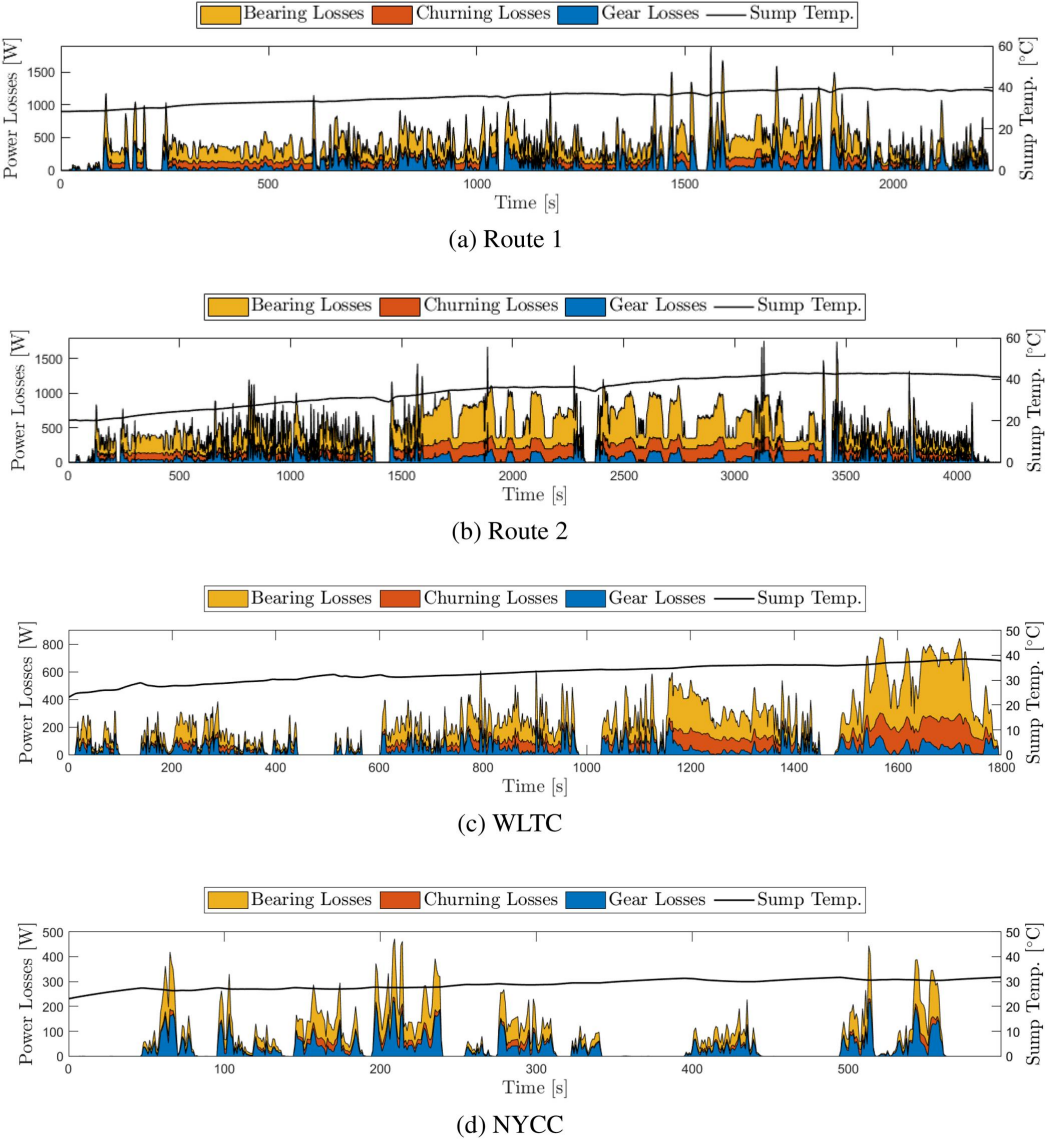
Area plots of gear (blue), churning (red) and bearing (yellow) loss evolution over different vehicle drive cycles. (a) Route 1 real-world drive cycle, (b) Route 2 real-world drive cycle, (c) light-duty vehicles test cycle (WLTC) class 3, and (d) New York City Cycle (NYCC).

The predicted losses from gear contact friction, bearings and gear churning over the cycles, are shown in Fig. 9. Combined power losses from all sources averaged 477 W, 618 W, 238 W, and 72 W over Route 1, Route 2, the WLTC, and NYCC, respectively. Gear meshing losses are seen to be high at periods when torque is high and speed is relatively low, such as when the vehicle accelerates from stationary. In contrast, gear churning losses only comprise a significant proportion of overall losses during periods when torque is low and speed is high, such as the period of cruising between 2,400 and 3,400 s in Fig. 9b (Route 2 cycle). Bearing losses, consisting of both load independent and load dependent losses, comprise the greatest proportion of overall losses for the majority of both of the real-world cycles and the WLTC. The analysis reveals remarkable differences in the relative importance of different loss sources in different drive cycles. For example, gear meshing friction is the second greatest contributor and gear churning the third in the lower speed Route 1 real-world cycle, but this ranking is reversed in the higher speed Route 2 and in the WLTC, with gear churning losses higher than gear mesh losses. This is likely due to the greater entrainment speeds in these cycles resulting in thicker lubricant films in the gear mesh and thus a reduction in effective mixed friction, coupled with greater gear churning due to extended periods of highway driving. Perhaps most strikingly, gear mesh losses are the largest source of power loss in the NYCC city cycle, significantly larger than bearing losses, in contrast to all other cycles analyzed here. This is the result of low film thickness (and hence high friction) in gear teeth contacts at low-speed (averaging approximately 11 km/h) and high torque vehicle conditions imposed by more frequent acceleration from standstill typical of city driving that is captured by NYCC. The effect of these loss differences between vehicle duty cycles on optimum lubricant properties is explored in the following section.

### Optimization of e-fluid viscosity

The EDU efficiency model described in this paper makes it possible to quantify the impact of changing a single fluid property on overall EDU power losses. Arguably the most important e-fluid property to optimize for improved EDU efficiency is the viscosity. In this section, a systematic study of the influence of fluid viscosity on EDU power losses is presented.

The real fluid whose properties are described in Table 4 was used as a baseline in this study. Its viscosity at 100 °C is 5.9 cSt. Eight hypothetical fluids with different viscosities were then generated by changing the kinematic viscosity at 100 °C (KV100) between 1 and 10 cSt, with the viscosity at 40 °C varied accordingly to keep the viscosity index (VI) the same in all cases. All other properties were kept the same as the baseline fluid. While in reality, other lubricant parameters, notably the pressure-viscosity coefficient α may also be expected to change with viscosity, (*59*) these dependencies have been intentionally neglected here to isolate the influence of viscosity on overall losses. In principle, the described approach makes it possible to study the influence of changing α. However, as the bearing loss model does not include α explicitly and given the significance of bearing losses, a study on its influence on overall transmission losses could lead to misleading results and so has been avoided here.

**Figure 10.**
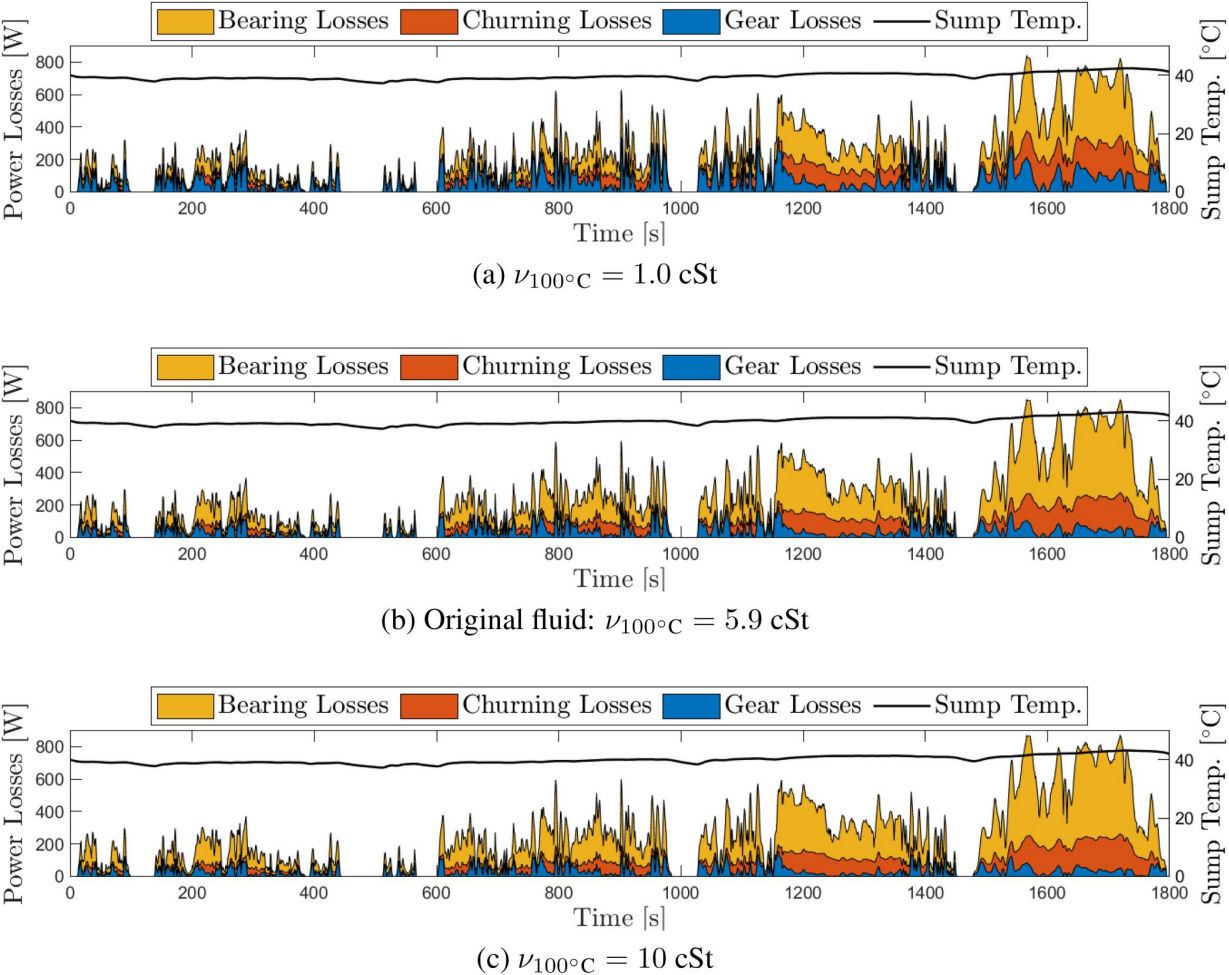
Area plots of transmission power losses over light-duty vehicles test cycle (WLTC) with three different fluid viscosities (a) hypothetical fluid $\nu_{100^{{}^{\circ}}C}=1$ cSt, (b) original baseline fluid $\nu_{100^{{}^{\circ}}C}=5.9$ cSt and (c) hypothetical fluid $\nu_{100^{{}^{\circ}}C}=10$ cSt. In all cases viscosity index (VI) = 174; initial transmission temperature is 40 °C and ambient temperature is 23 °C; calculations were done for six additional viscosities (not shown here).

Each of the hypothetical fluids were modeled to predict power losses over the class 3 WLTC. In each case, the initial gearbox temperature was set to 40 °C, representative of the quasi-steady state temperature of the sump under these operating conditions. The choice of this temperature is of obvious importance when assessing the effect of viscosity over a drive cycle given that viscosity is a strong function of temperature. The approach taken here of setting the initial temperature at a value that is representative of steady-state conditions means that the loss comparison is made once the vehicle has been driven for some time, as opposed to assuming a cold start for example. Plots of gear, churning, and bearing losses over the whole WLTC for the fluids at the two extremes (KV100 values of 1 cSt and 10 cSt), and the baseline fluid (KV100 =5.9 cSt), are shown in Fig. 10.

A clear trend is evident in these plots; reduction in viscosity leads to a significant increase in gear meshing losses, but a decrease in bearing losses. The reduction in gear losses with increasing viscosity is particularly evident in periods of acceleration and deceleration, while the reduction in bearing losses with reducing viscosity occurs primarily during periods of high-speed, such as the Extra-High section of the WLTC. This trend is primarily due to the fact that gears are rougher ($R_{\text{qc}}=$280 nm in this case) so mostly operate in the mixed lubrication regime where an increase in viscosity leads to a decrease in friction; in contrast, bearings mostly operate in the full-film regime where any further increase in viscosity leads to an increase in hydrodynamic friction.

**Figure 11.**
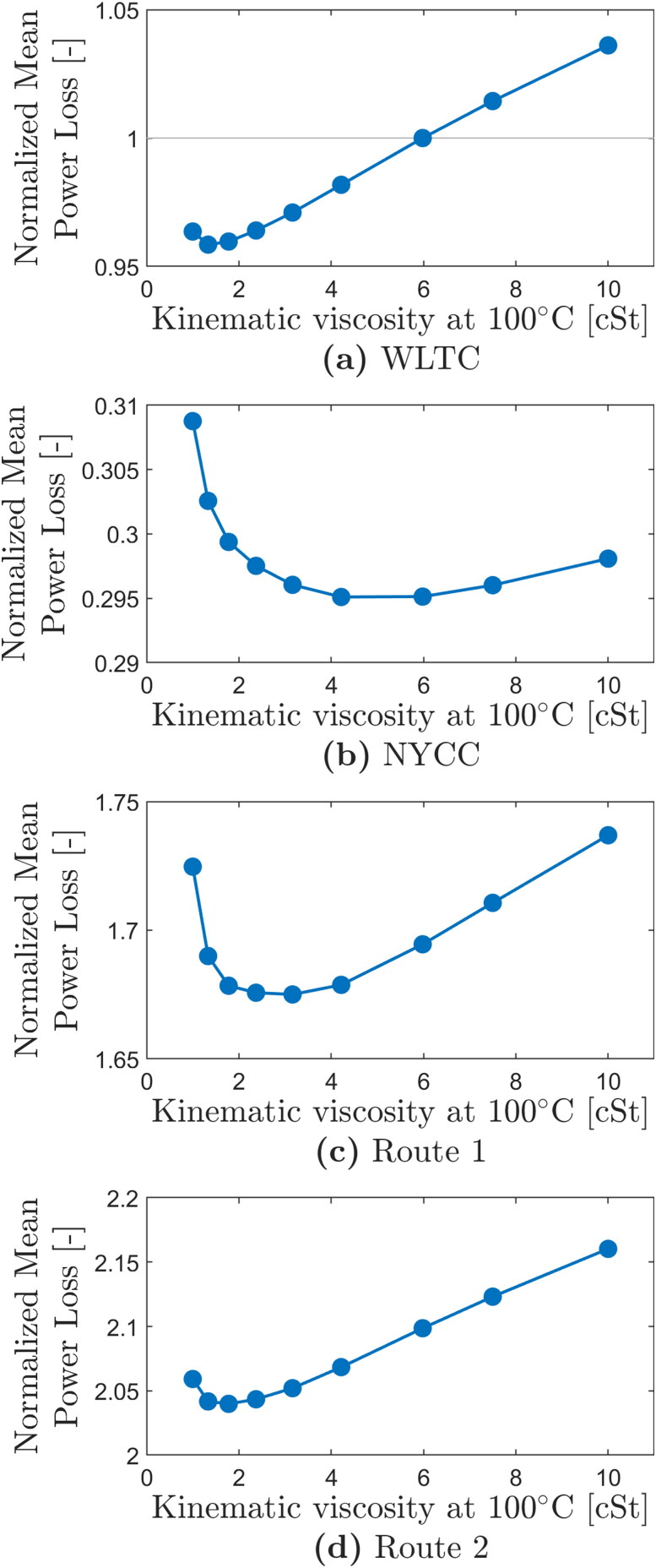
Mean power loss over a vehicle duty cycle (= Total energy loss in a cycle / total cycle time) plotted against e-fluid kinematic viscosity for four vehicle duty cycles: (a) Worldwide harmonized light vehicles test cycle (WLTC), (b) The New York City Cycle (NYCC), (c) Real-world drive cycle Route 1, and (d) Real-world drive cycle Route 2. In all cases mean power loss (y axis) is normalized by the magnitude of the mean power loss obtained with the baseline fluid with KV100=5.9 cSt in the WLTC (this makes it possible to compare power loss across the 4 cycles if of interest). In all cases viscosity index (VI) = 174. Initial transmission temperature is 40 °C and ambient temperature is 23 °C; corresponding power loss evolution over the WLTC is shown in Fig. 10 for three selected viscosities.

Fig. 11 plots the mean power loss over the WLTC, NYCC city cycle and the two real-world drive cycles against the e-fluid viscosity at 100 °C (KV100). The mean power loss is calculated as the total energy loss for each cycle divided by the total cycle time. Note that the mean power loss is used in this comparison instead of the total energy to make it possible to compare power losses across the four cycles; because the duration of each cycle is different the comparison of total energy loss across them would be meaningless. This figure serves to illustrate two key findings of this study: (i) For any given vehicle duty cycle, it is possible to determine an optimal e-fluid viscosity for which the overall vehicle power loss is minimum; and (ii) that this optimum e-fluid viscosity is very different for different vehicle duties.

For the low speed, frequent start-stop conditions of the NYCC city driving cycle the optimum KV100 viscosity is predicted to be around 5–6 cSt. Interestingly, this is in line with the viscosity of typical ATFs (Dexron VI) and e-fluids commonly used in today’s EVs. The optimum viscosity for the other 3 cycles is lower and decreases in the order Route 1 (mixture of urban and highway), Route 2 (mainly highway) and WLTC (light duty). For these particular conditions and model inputs, the optimum viscosity for the WLTC is predicted to be as low as 2 cSt (this viscosity produces approximately 4% lower transmission power losses than the baseline fluid of KV100 5.9 cSt). However, the WLTC is a relatively mild cycle in terms of transmission loads and speeds and it is obvious from the results for all other cycles that such low viscosity is unlikely to result in optimum efficiency for most vehicle operating conditions. Figure 11 also shows that the power loss curves are relatively flat near the optimum viscosity which means that viscosities slightly higher than the exact optimum values do not significantly change the power losses but may offer other benefits, not least improved surface protection. Perhaps more importantly, Fig. 11 also illustrates that reducing the viscosity to ultra-low values can be counter-productive and actually lead to an increase in power losses. This is because the gear tooth contact friction increases significantly with decreasing viscosity in this region. In the other extreme, increasing the viscosity past the optimum value leads to increasing bearing and gear churning losses similarly resulting in an increase in the overall losses. It must be stressed that the intention of this analysis is to illustrate trends in the effect of vehicle duty on optimum e-fluid viscosity; the values of these optimum viscosities depend on the exact conditions imposed, details of transmission design, and choices made for model inputs in these specific examples and thus should not be taken as general recommendations.

### Relative contributions of individual loss sources

**Figure 12.**
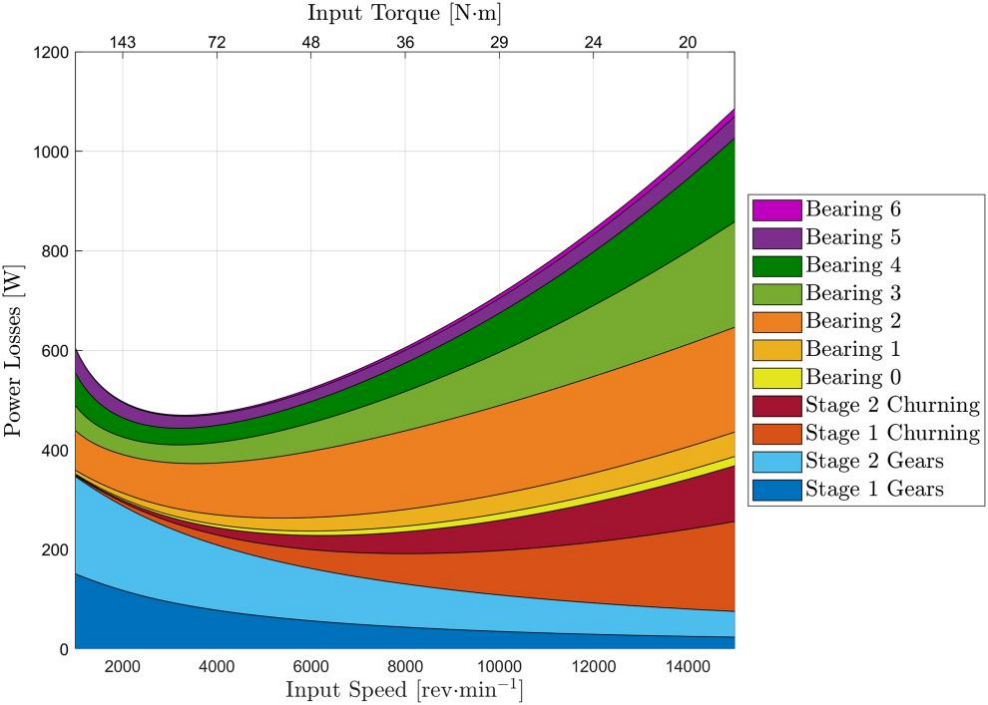
Breakdown of transmission power losses from 1000 to 15,000 rpm input speeds under constant 30 kW input power and 40${}^{{}^{\circ}}$C transmission temperature.

The losses over the complete drive cycle are useful when considering the practical impact of design choices on vehicle efficiency. However, due to the transient speeds and torques imposed on the transmission, they do not lend themselves well to analyzing the relative contribution of each loss source. Such analysis may be useful to help identify where the further development effort should be focused for maximum benefit in terms of vehicle efficiency. To analyze the relative contribution of each loss source, a simple case of a fixed input power to the transmission but increasing speed (and therefore decreasing torque) was considered. To further simplify the interpretation of results this analysis was done under isothermal conditions with a single fixed temperature for the sump and all parts of the transmission. The results of this analysis for the constant input power of 30 kW for the modeled EV transmission are shown in Fig. 12. The transmission temperature is fixed at 40 °C. Analyses at other temperatures and input powers produce similar overall trends in terms of the relative contributions of different loss sources under different speed/torque conditions, even if their absolute values may be different. The transmission input speed (motor speed) is shown on the main *x*-axis and the corresponding input torque for the fixed 30 kW power is shown on the secondary *x*-axis on top of the plot. As an indication of the vehicle conditions imposed here, given the specific characteristics of the modeled EV, 30 kW power at a gearbox input speed of 14,000 rpm may represent the vehicle cruising at 150 km·h${}^{-1}$ on a flat road; whereas, 30 kW at a gearbox input speed of 2,000 rpm is representative of the unladen vehicle driving at around 20 km·h${}^{-1}$ up an approximately 30% incline, for example.

Figure 12 clearly shows that gear tooth friction losses are the most significant loss source at low speeds, representing more than 50% of the overall losses at input speeds less than about 3,000 rpm. However, they decrease rapidly as the speed increases so that the relative contribution to overall loss at high speeds is small. This is because the oil film thickness in gear teeth contacts increases as transmission speed increases such that the gear teeth contacts, which usually operate in mixed lubrication, are pushed down the Stribeck curve closer to the full-film regime, hence reducing contact friction. In contrast, the gear churning losses only become significant at higher speeds. Bearing losses increase with speed (due to increasing bearing drag losses, that is, oil churning in bearings) but are significant across the speed range. The largest bearing loss is predicted to be that from Bearing 2 which is the highly loaded input shaft bearing which is set to carry input shaft axial load in the present transmission architecture. The losses in the intermediate shaft bearings (Bearings 3 and 4) are also significant, while those on the low-speed output shaft are small.

## Discussion

The method presented in this paper provides a tool to quickly and economically evaluate the effect of e-fluid properties and transmission design on overall EV transmission efficiency. Gear mesh friction, gear churning and bearing losses are all accounted for. The system-based approach captures the interaction between these loss sources by predicting the dynamic changes in transmission temperature during driving and the resultant influence of this on each of the losses during a drive cycle. The model utilizes measured lubricant rheology and boundary friction, allowing it to discriminate between two nominally the same specification fluids in terms of their impact on transmission losses. The method was validated by comparing its temperature predictions against measurements made on a popular EV during real-word drive cycles.

Vehicle operating conditions directly influence the relative contribution of individual losses: under high-torque, low-speed conditions, gear tooth friction losses represent the largest proportion of the overall losses because under these conditions lubricant film thickness in gear teeth contacts is relatively low which, combined with relatively high gear tooth roughness, means that gears operate deep in the mixed lubrication regime with significant metal-to-metal contact and thus high friction; at higher speeds, lubricant film thickness increases hence reducing gear teeth friction, such that gear losses become less important than bearing and churning losses. In general, bearing losses increase with speed but are significant throughout the speed range. They are dominated by losses in the high-speed input shaft bearing that carries the axial load in the present design. Intermediate shaft bearing losses are also significant across the speed and torque range, due to relatively large load-dependent losses and load-independent drag losses. Although the frictional moment on the output shaft bearings may be high due to their high loads, this does not translate to a significant proportion of overall power losses due to their comparatively low speed. Gear churning losses are only significant at high speeds. These insights into loss breakdown can help to explain the observed trends seen in the optimum viscosity study for different vehicle duty cycles. A higher oil viscosity is likely to reduce gear losses during low-speed high-torque operation (such as frequent start-stop operation as in the NYCC city cycle), but will increase bearing drag and churning losses during high-speed operation (such as during highway cruising). This means that in general, a car driven mainly in a city will benefit from a higher e-fluid viscosity than one being mainly driven on a highway. For example, the model predicts the optimum viscosity for max efficiency in the NYCC cycle to be between 5 and 6 cSt, while for Route 2 real-world experimental cycle, which includes mainly highway driving, its optimum viscosity is predicted to be lower at 2–3 cSt. Each optimum viscosity depends strongly on the exact torque-speed profile of any given drive cycle as shown in this paper. However, the trends also show that reducing viscosity to ultra-low values, beyond the identified optimums, can have a negative effect of increasing the overall power losses due to the described interplay between gear, churning and bearing losses.

### Model limitations and proposed improvements

Although the model was shown to be accurate when its predictions were compared to real world measurements, it includes a number of limitations. Chief amongst these is the simplistic treatment of the e-motor losses. These are simply taken from a published efficiency map of the relevant e-motor at one given temperature. The effect of these losses on the e-fluid temperature evolution, and hence its effect on the various loss sources, is included in the model. However, the effect of evolution of temperature on the motor losses themselves is ignored. E-motor efficiency is known to be a function of temperature so this is an important omission. As the transmission and e-motor form an integrated system, with the e-fluid playing a crucial role in e-motor cooling, application of the model to optimize the e-fluid, particularly its thermal properties, should ideally consider this temperature dependence of e-motor losses. This calls for an integrated approach to optimizing the overall EV powertrain efficiency considering both the e-motor and the transmission losses. If it was to become available, a full model of e-motor efficiency could easily be integrated into the present model. A simple approximate way of dealing with this is to characterize e-motor losses at a range of temperatures and use these data to estimate the motor losses at any given predicted EDU temperature.

The second major deficiency of the model lies in its treatment of bearing losses. In the current approach, bearing losses are predicted using publicly available semi-empirical models. (*48,50,60*) The original version of this bearing friction model (*48,60*) is stated by its authors to overestimate bearing drag losses at higher bearing speeds so should not be used for bearing speeds of $n\cdot d_{m}>0.5\times 10^{6}$ rpm·mm. This limit is often exceeded in a typical EV transmission owing to high input speeds. In an attempt to address this, the present approach implements the updated bearing drag loss predictions proposed by Morales-Espejel and Wemekamp. (*50*) However, several deficiencies in the implemented bearing loss predictions persist. One of these is the accurate determination of immersion depth for each of the bearings, particularly those on the high-speed input shaft where drag losses may be significant. In the modeled transmission, the input shaft bearings are above the nominal sump level and therefore rely on the distribution of oil from splashing from the gears and from the oil spray. For this reason, the immersion depth of the input shaft bearings was assumed to be zero in the presented results so that drag losses are effectively neglected for this shaft. In reality, the immersion depth of these, and other bearings in the present EDU, will be determined by the distribution of the oil in the casing which may be expected to dynamically change with varying vehicle speed as the gears throw the oil around the gearbox; this may have an important impact on bearing drag losses and cooling. Another important limitation of the simple bearing loss model used here is the simplification of the role of oil rheology; this is only accounted for by implementing different constant full-film COF values depending on whether the test fluid is broadly characterized as a mineral, synthetic or transmission fluid (*48,60*); viscosity is the only fluid property considered directly, while important rheological properties of the fluid, not least the pressure-viscosity coefficient, are not considered directly. While for many engineering applications this may be sufficient, it limits the utility of the model when comparing the performance of nominally similar oils. This is particularly important as bearings were shown to represent a large proportion of overall losses for many driving conditions. It should be noted that while the simple bearing loss model implemented here, which is taken from published literature, suffers from the described limitation in the treatment of oil rheology, bearing manufacturers may well possess more advanced, proprietary models able to better account for oil rheology.

## Conclusions

This paper describes a new system-level, thermally-coupled approach to predict power losses in an EV transmission. The method considers gear, bearing and churning losses and takes into account interaction between them by predicting temperature evolution over an entire drive cycle. The model can be used as a fast and efficient tool for optimization of e-fluid properties and transmission design. The model is then used to illustrate the relative contributions of different loss sources for different vehicle duties and to assess the impact of e-fluid viscosity on the overall losses. The main conclusions may be summarized as follows:

- Gear friction is predicted using a thermally coupled iterative procedure to account for coupling between gear teeth contact friction and film thickness via evolution of gear tooth temperature. The approach predicts mixed regime friction using a function dependent on full film friction, boundary friction, and lambda ratio. The model is applied at multiple points along the path of contact accounting for the changing contact conditions resulting from the involute tooth geometry and load sharing between tooth pairs.
- The gear tooth friction model utilizes experimentally derived rheological data and measured boundary friction for each considered fluid. This allows the model to discriminate between nominally the same specification lubricants in terms of their impact on transmission efficiency.
- Gear churning losses are predicted with a newly derived empirical model which accounts for the influence of viscosity on the dynamic oil surface profile and is applicable to high-speeds. The bearing losses are predicted using an existing bearing friction model devised by other authors.
- EDU temperature evolution over time is predicted using a thermal network approach, accounting for additional heat from the e-motor and cooling via a heat exchanger. This crucial step enables the model to account for the thermal interaction between the various sources of power losses in the transmission.
- Model predictions for temperatures at various nodes within an EDU are compared to corresponding measurements made on a popular EV for a series of experimental drive cycles. Good agreement was shown for all nodes and drive cycles.
- Gear friction losses are significant at low speed/high torque conditions but are only a small proportion of the overall loss at high speeds, while gear churning is only significant at high speeds. Bearing losses increase with speed but are important for all conditions; they are dominated by bearing losses in the input shaft bearing that carries the axial load as well as the friction and drag losses in the intermediate shaft bearings.
- There exists an optimum oil viscosity that minimizes the overall power loss for a given vehicle duty cycle.
- The exact value of this optimum viscosity is highly dependent on the torque-speed characteristics of the given drive cycle; for the NYCC city driving cycle, which has low average speed and contains frequent start-stops, the optimum viscosity is shown to be 5–6 cSt at 100 °C, but this is lower for highway driving and the relatively light duty WLTC.
- For all vehicle duties analyzed here, continuous decrease in viscosity to ultra-low values can be counterproductive and lead to an increase in overall transmission losses.

## Figures and Tables

**Figure B1.**
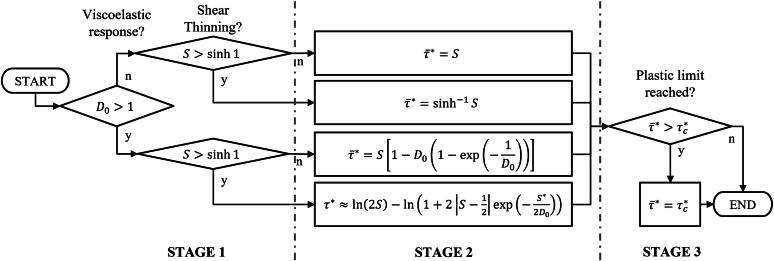
Flowchart used to determine dimensionless mean shear stress, based on procedure described by Olver and Spikes. (*35*)

**Figure C1.**
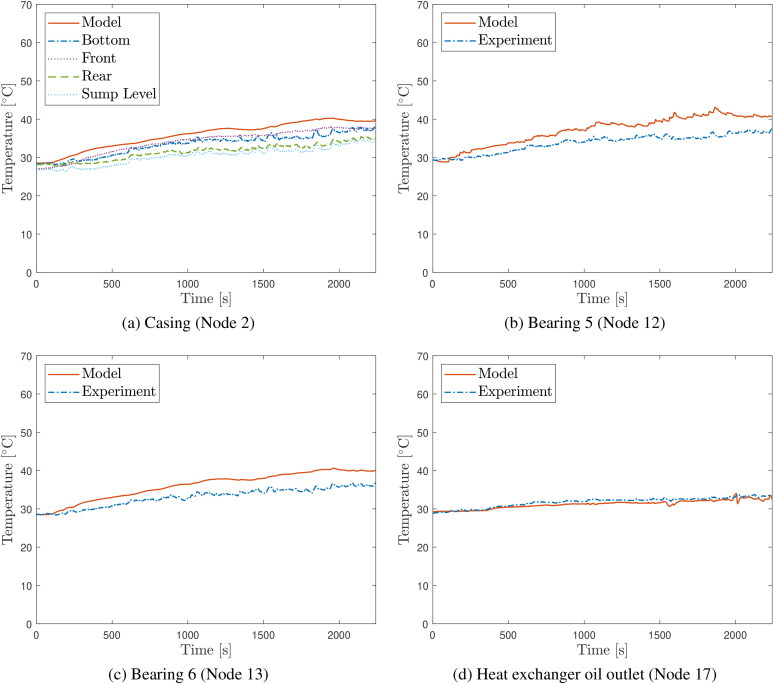
Comparison of temperature predictions to thermocouple measurements over the Route 1 road test at four different nodes in the gearbox.

**Figure C2.**
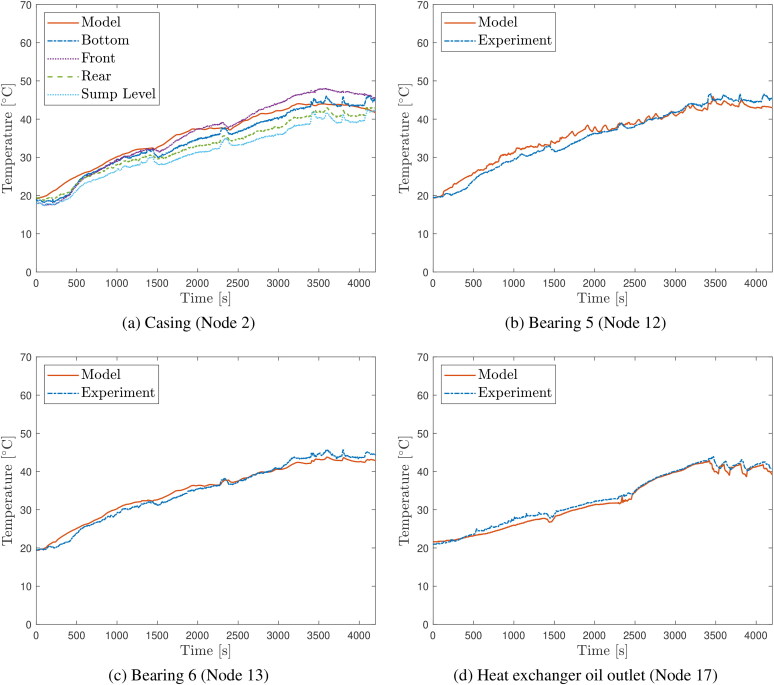
Comparison of temperature predictions to thermocouple measurements over the Route 2 road test.

## Appendices Appendix A

Descriptions of the nodes used for the modeled EDU are collated in Table A1.

**Table A1.** Thermal network nodes. (*47*)

Node	Description	Boundary condition?	Heat source
1	Ambient air	Yes	–
2	Casing	No	–
3	Stator casing	No	–
4	Rotor shaft	No	Motor losses & 1${}^{\text{st}}$ gear stage
5	Intermediate shaft	No	1${}^{\text{st}}$ & 2${}^{\text{nd}}$ gear stages
6	Output shaft	No	2nd gear stage
7	Bearing 0	No	Bearing 0 losses
8	Bearing 1	No	Bearing 1 losses
9	Bearing 2	No	Bearing 2 losses
10	Bearing 3	No	Bearing 3 losses
11	Bearing 4	No	Bearing 4 losses
12	Bearing 5	No	Bearing 5 losses
13	Bearing 6	No	Bearing 6 losses
14	Oil sump	No	Churning losses
15	Oil in motor annulus	No	–
16	Stator	No	Motor losses
17	Heat exchanger oil output	Yes	–

Equation [1] can be expressed in terms of the thermal resistances between each node, the temperatures of each node, and the heat into each node, with Eq. [11]:

[11] $$\begin{array}{c} \left[\begin{array}{c} Q_{1}\\ Q_{2}\\ \vdots \\ Q_{n} \end{array}\right]=\left[\begin{array}{cccc} \sum_{i=1}^{n}\left(\frac{1}{R_{{\text{th}}_{1,i}}}\right) & -\left(\frac{1}{R_{{\text{th}}_{1,2}}}\right) & \cdots & -\left(\frac{1}{R_{{\text{th}}_{1,n}}}\right)\\ -\left(\frac{1}{R_{{\text{th}}_{2,1}}}\right) & \sum_{i=1}^{n}\left(\frac{1}{R_{{\text{th}}_{2,i}}}\right) & \cdots & -\left(\frac{1}{R_{{\text{th}}_{2,n}}}\right)\\ \vdots & \vdots & \ddots & \vdots \\ -\left(\frac{1}{R_{{\text{th}}_{n,1}}}\right) & -\left(\frac{1}{R_{n,2}}\right) & \cdots & \sum_{i=1}^{n}\left(\frac{1}{R_{{\text{th}}_{n,i}}}\right) \end{array}\right]\left[\begin{array}{c} T_{1}\\ T_{2}\\ \vdots \\ T_{n} \end{array}\right]+\left[\begin{array}{c} {\left(mc_{p}\frac{\text{dT}}{\text{dt}}\right)}_{1}\\ {\left(mc_{p}\frac{\text{dT}}{\text{dt}}\right)}_{2}\\ \vdots \\ {\left(mc_{p}\frac{\text{dT}}{\text{dt}}\right)}_{n} \end{array}\right] \end{array}$$

where $R_{\left(i,j\right)}$ is the thermal resistance between nodes *i* and *j* and $T_{i}$ is the temperature of node *i*. By convention, when i=j, $1/R_{\left(i,j\right)}=0\text{.}$

## Appendix B

Olver and Spikes’ (*35*) algorithm for determining the dimensionless mean shear stress in the gear mesh is illustrated in Fig. B1. This requires the calculation of three dimensionless parameters: the Deborah number $D_{0}\text{,}$ the dimensionless strain rate *S* and the dimensionless limiting shear stress $\tau_{c}^{*}\text{,}$ defined by Eqs. [12], [13], and [14], respectively:

[12] $$D_{0}=\frac{\eta_{0}U}{2aG_{e}}$$

[13] $$S=\frac{\dot{\gamma }\eta_{0}}{\tau_{0}}$$

[14] $$\tau_{c}^{*}=\frac{\tau_{c}}{\tau_{0}}$$

where *a* is the Hertzian semiwidth, $G_{e}$ is the effective elastic shear modulus, *U* is the entrainment speed, and $\tau_{c}$ is the critical shear stress.

## Appendix C

Figures C1 and C2 show comparisons between model temperature predictions and experimentally measured temperatures for thermal network nodes 2, 12, 13 and 17 over the Route 1 and Route 2 real-world road tests, respectively.

## Nomenclature

a: Hertzian semiwidth [m]
b: Facewidth [m]
$C_{\text{ch}}$: Churning torque [N·m]
$c_{p}$: Specific heat capacity [J·kg${}^{-1}$K${}^{-1}$]
$D_{0}$: Deborah number [-]
$d_{m}$: Mean bearing diameter [m]
Fr: Froude number ($\text{Fr}=\omega^{2}R_{p}/g$)
G: Elastic shear modulus [Pa]
g: Gravitational acceleration (9.8 m·s${}^{-2}$)
$h_{0}$: Nominal gear immersion depth [m]
$h_{\text{eff}}$: Effective gear immersion depth [m]
m: Mass [kg]
n: Bearing rotational speed [rpm]
$\bar{p}$: Mean Hertzian contact pressure [Pa]
$p_{r}$: Reference pressure (1.96×10^8^ Pa)
Q: Heat [W]
Re: Reynolds number ($\text{Re}=\omega R_{p}^{2}/\nu$)
$R_{a}$: Gear addendum radius [m]
$R_{p}$: Gear reference radius [m]
$R_{\text{th}}$: Thermal resistance [K·W${}^{-1}$]
S: Dimensionless strain rate [-]
$S_{0}$: Atmospheric slope index [-]
T: Temperature [K]
$T_{r}$: Reference temperature [K]
t: Time [s]
U: Entrainment speed [m·s${}^{-1}$]
z: Roelands parameter [-]
$\dot{\gamma }$: Strain rate [s${}^{-1}$]
$\delta_{r}$: Gear radial clearance [m]
$\delta_{x}$: Gear axial clearance [m]
$\eta_{0}$: Inlet dynamic viscosity [Pa·s]
$\eta_{p}$: In-contact dynamic viscosity [Pa·s]
$\eta_{r}$: Reference viscosity [Pa·s]
$\eta_{\infty }$: Viscosity constant (6.31×10${}^{-5}$ Pa·s)
Λ: Specific film thickness [-]
μ: Mixed regime COF [-]
$\mu_{b}$: Boundary COF [-]
$\mu_{f}$: Full-film EHL COF [-]
ν: Kinematic viscosity [m^2^s ${}^{-1}$]
ρ: Density [kg·m${}^{-3}$]
τ: Shear stress [Pa]
${\bar{\tau }}^{*}$: Dimensionless mean shear stress [-]
$\tau_{0}$: Eyring stress [Pa]
$\tau_{c}$: Critical shear stress [Pa]
ϕ: Casing fill ratio [-]
ω: Rotational speed [rad·s${}^{-1}$]
